# Causal relationship between blood metabolites and risk of five infections: a Mendelian randomization study

**DOI:** 10.1186/s12879-023-08662-6

**Published:** 2023-10-07

**Authors:** Zhengxiao Wei, Qingqing Xiong, Dan Huang, Zhangjun Wu, Zhu Chen

**Affiliations:** 1 Department of Clinical Laboratory, Chengdu Public Health Clinical Medical Center & Public Health Clinical Center of Chengdu University of Traditional Chinese Medicine, 377 Jingming Road, Jinjiang District, Chengdu, 610066 China; 2 Department of Scientific Research and Teaching, Chengdu Public Health Clinical Medical Center & Public Health Clinical Center of Chengdu University of Traditional Chinese Medicine, 377 Jingming Road, Jinjiang District, Chengdu, 610066 China

**Keywords:** Blood metabolites, Mendelian randomization, Infections, Sepsis, Pneumonia, Urinary tract infection

## Abstract

**Objective:**

Infectious diseases continue to pose a significant threat in the field of global public health, and our understanding of their metabolic pathogenesis remains limited. However, the advent of genome-wide association studies (GWAS) offers an unprecedented opportunity to unravel the relationship between metabolites and infections.

**Methods:**

Univariable and multivariable Mendelian randomization (MR) was commandeered to elucidate the causal relationship between blood metabolism and five high-frequency infection phenotypes: sepsis, pneumonia, upper respiratory tract infections (URTI), urinary tract infections (UTI), and skin and subcutaneous tissue infection (SSTI). GWAS data for infections were derived from UK Biobank and the FinnGen consortium. The primary analysis was conducted using the inverse variance weighted method on the UK Biobank data, along with a series of sensitivity analyses. Subsequently, replication and meta-analysis were performed on the FinnGen consortium data.

**Results:**

After primary analysis and a series of sensitivity analyses, 17 metabolites were identified from UK Biobank that have a causal relationship with five infections. Upon joint analysis with the FinGen cohort, 7 of these metabolites demonstrated consistent associations. Subsequently, we conducted a multivariable Mendelian randomization analysis to confirm the independent effects of these metabolites. Among known metabolites, genetically predicted 1-stearoylglycerol (1-SG) (odds ratio [OR] = 0.561, 95% confidence interval [CI]: 0.403–0.780, *P* < 0.001) and 3-carboxy-4-methyl-5-propyl-2-furanpropanoate (CMPF) (OR = 0.780, 95%CI: 0.689–0.883, *P* < 0.001) was causatively associated with a lower risk of sepsis, and genetically predicted phenylacetate (PA) (OR = 1.426, 95%CI: 1.152–1.765, *P* = 0.001) and cysteine (OR = 1.522, 95%CI: 1.170–1.980, *P* = 0.002) were associated with an increased risk of UTI. Ursodeoxycholate (UDCA) (OR = 0.906, 95%CI: 0.829–0.990, *P* = 0.029) is a protective factor against pneumonia. Two unknown metabolites, X-12407 (OR = 1.294, 95%CI: 1.131–1.481, *P* < 0.001), and X-12847 (OR = 1.344, 95%CI: 1.152–1.568, *P* < 0.001), were also identified as independent risk factors for sepsis.

**Conclusions:**

In this MR study, we demonstrated a causal relationship between blood metabolites and the risk of developing sepsis, pneumonia, and UTI. However, there was no evidence of a causal connection between blood metabolites and the risk of URTI or SSTI, indicating a need for larger-scale studies to further investigate susceptibility to certain infection phenotypes.

**Supplementary Information:**

The online version contains supplementary material available at 10.1186/s12879-023-08662-6.

## Introduction

Infections have long been recognized as a global public health priority, which account for over 20% of deaths worldwide [1]. From 2009 to 2013, infections affected around one-fourteenth of the global population, thus amplifying the burden of disease globally [2]. Due to antibiotic resistance, aging populations, and the emergence of new pathogens, the burden of disease is expected to increase. Therefore, identifying modifiable risk factors for these infections is crucial.

With the advent of high-throughput technology, we are now able to measure hundreds of circulating metabolites and perform gene typing in large-scale populations in parallel. By aggregating the statistics from Shin et.al, various metabolic characteristics have been found to have chance associations with the risks of several diseases, such as cardiovascular disease, autoimmune disease, polycystic ovary syndrome, and mental illness [3–5]. However, evidence for the discussion of blood metabolites and the risk of infections is lacking, even though some studies have described several modifiable risk factors (such as body mass index, body fat percentage, total cholesterol level, and low-density lipoprotein-cholesterol) [6, 7]. Given the intrinsic limitations of traditional observational research, an unequivocal metabolic spectrum that contributes to infectious diseases based on existing evidence cannot be provided.

Mendelian Randomization (MR) is an increasingly prevalent analytical technique that has been extensively employed to deduce the causal impact of exposures on outcomes [8]. In the absence of a randomized controlled trial (RCT) or the initiation of a new RCT, the approach is an important alternative strategy that can provide reliable evidence of a causal relationship between exposure and disease risk [9].

In the current study, we aimed to adopt an approach to determine the potential causal impaction of blood metabolites on the risk of five infections. We selected five infection phenotypes with a relatively high incidence in Europe: sepsis, pneumonia, upper respiratory tract infection (URTI), urinary tract infection (UTI), and skin and subcutaneous tissue infection (SSTI) [6, 10]. Meanwhile, these selections were also based on their possessing adequate sample sizes to carry out a GWAS with enough power. This study used a full-exposure design containing more than 400 blood metabolites to provide reliable support for the establishment of feasible infectious disease screening and prevention strategies in clinical practice.

## Methods

### Study design

**Fig. 1:**
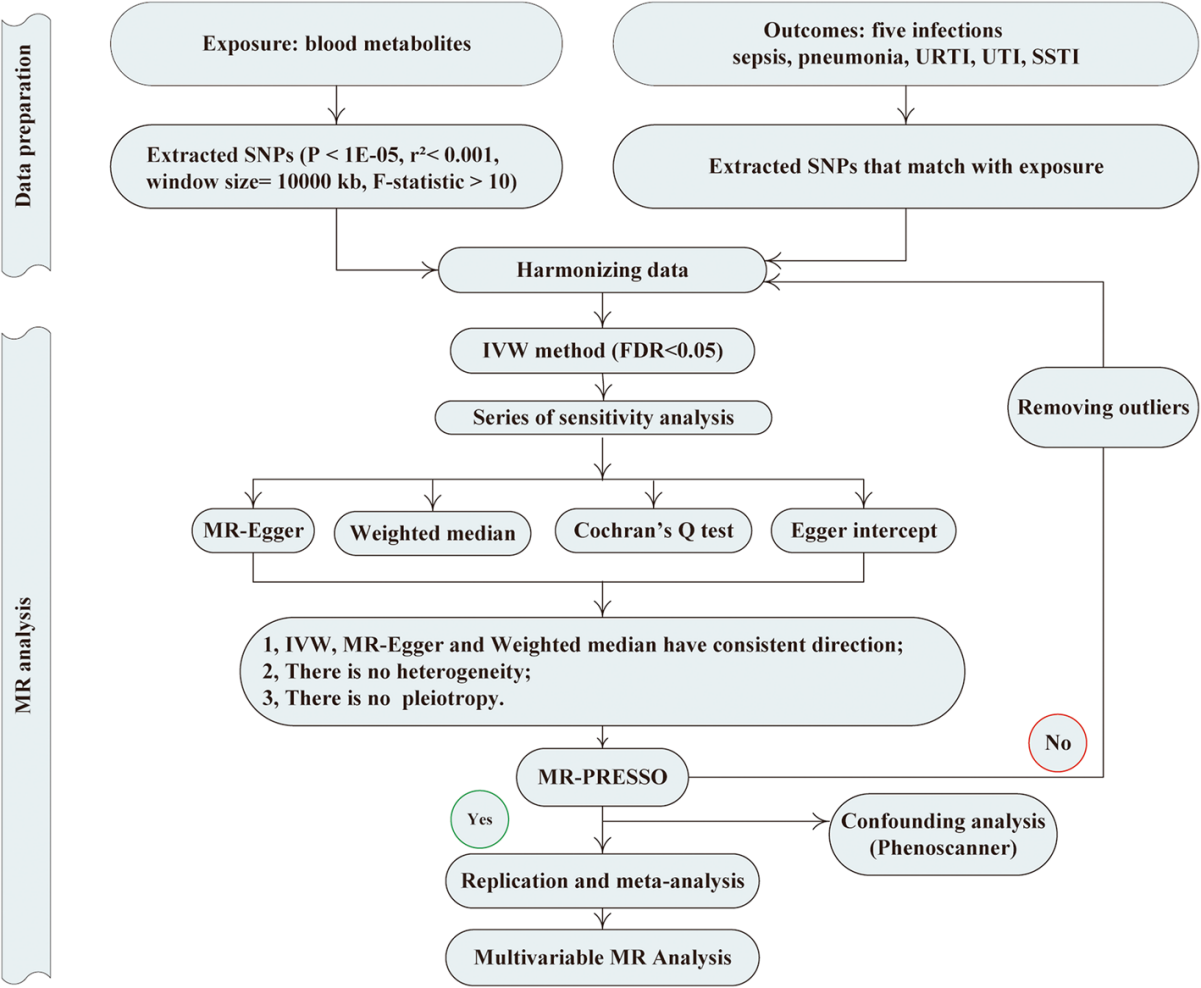
Flow chart for the Mendelian randomized analysis. IVW, inverse variance weighted; URTI, upper respiratory tract infection; UTI, urinary tract infection; SSTI, skin and subcutaneous tissues

We conducted multiple two-sample studies to systematically evaluate the intrinsic connections in the range of 452 blood metabolites to the occurrence risk of five infections. Assuming multiple cohorts share similarities in epidemiology and genetics, employing genome-wide significant associations within a larger cohort can efficiently amplify sample size, elevate the potential to detect rare associations, and enhance statistical power. Therefore, our analysis was bidirectional, beginning with an assessment of the causal impact of metabolites on five infection phenotypes, followed by an investigation into the reverse relationship. To ensure the credibility of the design, we also carried out a series of statistical methods to test the results. Summary data on infections was collected from two separate GWAS databases, with the UK Biobank cohort [11] utilized for preliminary analysis and a range of sensitivity tests, while data from the FinnGen cohort [12] was used for replication analysis, and meta-analysis was conducted to strengthen the results. A summary of the study design and data sources can be found in Fig. 1 and Table 1.

**Table 1:** Source of outcome genome-wide association study summary data

Outcome	Source	Cases	Control	GWAS ID/Phenocode	Population
Sepsis	UK Biobank	10154	454764	ieu-b-69	European
	FinnGen Mrach 2023 Release	10666	303314	AB1_other_sepsis	European
Pneumonia	UK Biobank	22576	463917	ieu-b-4976	European
	FinnGen Mrach 2023 Release	52021	290478	J10_pneumoni	European
URTI	UK Biobank	2795	483689	ieu-b-5063	European
	FinnGen Mrach 2023 Release	61543	280956	J10_UPPERINFEC	European
UTI	UK Biobank	21958	464256	ieu-b-5065	European
	FinnGen Mrach 2023 Release	2588	163702	N14 Urethraoth	European
SSTI	UK Biobank	4035	357159	L08 other local infections of skin and subcutaneous tissue	European

*Abbreviations*: *URTI* upper respiratory tract infection, *UTI* urinary tract infection, *SSTI* skin and subcutaneous tissue infection

### Selection of metabolite genetic instruments

The GWAS data on blood metabolites were obtained from the Metabolomics GWAS server (http://metabolomics.helmholtz-muenchen.de/gwas/), which included genetic information from 7,824 individuals of European ancestry. Genome-wide association scans and high-throughput metabolomic analyses detected approximately 2.1 million SNPs and 452 metabolites [13], of which the chemical properties of 177 metabolites have yet to be determined. Based on the Kyoto Encyclopedia of Genes and Genomes (KEGG) database, the remaining 275 metabolites were chemically recognized and classified into eight major metabolic categories, which include xenobiotics, nucleotides, amino acids, lipids, cofactors and vitamins, energy, peptides, and carbohydrates [14].

Qualified genetic variants associated with metabolites were selected through a series of steps. First, given the scarcity of SNPs reaching genome-wide significance, we eased the criteria and adopted a *P* < 1E-05 cutoff to obtain the top independent SNPs (r^2^ < 0.001 within 10,000-kb windows), consistent with Yang et al.'s study [15]. This method has been widely used in previous MR studies [16]. At the same time, to avoid bias arising from weak instrumental variables, we calculated the F-statistic for each SNP to measure statistical strength. SNPs with F > 10 were selected for further analysis because they are unlikely to be weak instrumental variables.

We obtained exposure SNPs by extracting them from the results and removed outcome-related SNPs (*P* < 1E-05). Missing SNPs in the outcome were discarded. An allele calibration process was executed for harmonization purposes to ensure that exposure- and outcome- SNPs were in alignment, with the exclusion of palindrome SNPs with intermediate effect allele frequency (EAF > 0.42) or incompatible alleles (such as A/G and A/C). Lastly, we retained only those metabolites that had three or more SNPs for MR analysis.

### GWAS data for infection outcomes

We conducted MR analysis on five infection phenotypes (Table 1): sepsis, pneumonia, upper respiratory tract infection (URTI), urinary tract infection (UTI), and skin and subcutaneous tissue infection (SSTI). These were selected because there was a sufficient sample size to perform a powerful GWAS. The GWAS results for all of these diseases came from two independent European ancestry cohort databases: the UK Biobank [11] and FinnGen Release 8 [12]. To determine whether genetic variation is associated with common infection phenotypes, we used the UKB cohort, which has whole-genome gene typing data. We then extracted these summary data from the GWAS analysis in the latest version of the UK Biobank Infectious Disease Genetics Project. In order to confirm the validity of our findings via replication and meta-analysis, we made use of data from the FinnGen consortium, which defines cases and controls using ICD-10 codes obtained from digital health records, and these data are publicly available on their website: https://r8.fnngen.f/pheno.

### MR preliminary analysis

The primary strategy utilized in this study to evaluate the initial connection between metabolites and infections was the inverse variance weighted (IVW) method. IVW is a method widely used in MR Research that estimates causal effects by weighting the Wald ratios of each SNP [17]. IVW is one of the most important MR estimation methods, it assumes that all genetic variants are valid, which may be susceptible to pleiotropic bias. Therefore, supplementary methods, including weighted median (WM) and MR-Egger (slope-intercept), were also employed in this study. WM, as a robust MR method, can still offer stable estimates even when more than half of the information sourced from invalid IVs [18], while MR-Egger regression can identify and correct for potential pleiotropy, providing estimates that are relatively consistent [19].

### MR sensitivity analysis

A sensitivity analysis was conducted to assess potential biases in the MR hypothesis after identifying significant estimates (IVW *P* < 0.05). The presence of heterogeneity was detected by using Cochran’s Q test [20], which yielded a *P* value of < 0.05 and an I^2^ value of > 25%. The level of horizontal pleiotropy was assessed by the Egger intercept [19]. The MR-PRESSO test was utilized to identify outliers [21], meanwhile, in order to detect individual SNP that had a significant impact on merged IVW estimates, a leave-one-out (LOO) analysis was carried out. Finally, the MR Steiger test was performed to confirm the directionality of the association for the five infections [22]. The false discovery rate method was used to correct multiple testing. To be considered statistically significant, a metabolite’s estimated causal effect had to have a Benjamini–Hochberg adjusted *P*-value less than 0.05. *P*-values that were originally < 0.05 but had adjusted *P*-values > 0.05 were suggestive of correlation.

We subsequently investigated the potential association of the SNPs related to metabolites with other phenotypes through the utilization of the PhenoScanner V2 website (http://www.phenoscanner.medschl.cam.ac.uk/). SNPs that exhibited associations with potential confounding factors, such as body mass index, body fat percentage, total cholesterol level, and low-density lipoprotein cholesterol, were removed, and IVW was carried out again to confirm the robustness of the results.

Assessing the metabolites’ causal effects on infections via diverse MR methods.

In order to reinforce the strength and credibility of the selected metabolites, we replicated the IVW analyses using the GWAS data from the FinnGen consortium, employed the METAL [23] (version 201,103–25) to execute a meta-analysis of available GWAS data from the FinnGen Consortium and UK Biobank for each of the infection phenotypes. The meta-analysis was executed to identify a set of candidate metabolites for our subsequent multivariable Mendelian randomization analysis. We then performed a reverse Mendelian randomization analysis, utilizing the disease as the exposure and the metabolites as the outcome, to explore whether a reverse causal relationship exists between the identified metabolites and the disease.

#### Statistical analysis

All statistical analyses were performed using R software (version 4.2.3). For the univariable Mendelian randomization analysis, the “TwoSampleMR” package was employed, while the “Mendelian Randomization” and “MVMR” packages in R were utilized for multivariable MR analysis in this study. METAL [23] (version 2011–03-25) was used to perform the meta-analyses of the outcomes.

## Results

**Fig. 2:**
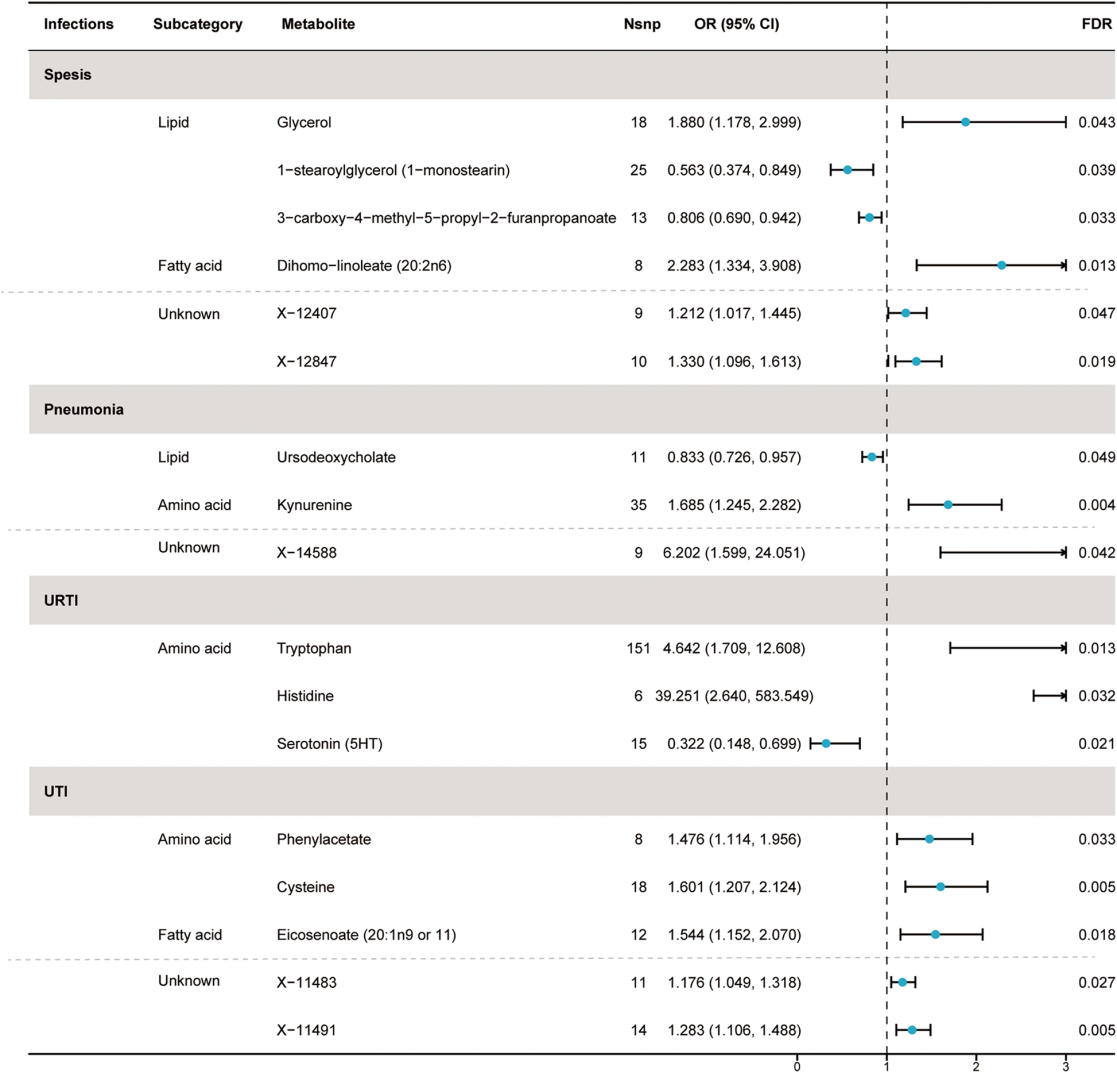
Forest plot for the causal effect of identified metabolites on the risk of 4 types of infection phenotypes (sepsis, pneumonia, URTI, and UTI) derived from inverse variance weighted (IVW). SNP, single nucleotide polymorphism; OR, odds ratio; CI, confidence interval; FDR, false discovery rate; URTI, upper respiratory tract infection; UTI, urinary tract infection

Based on preliminary instrument selection, the number of instrumental variables for metabolites ranged from 3 to 148, with a median of 13. Using these instrumental variables, we initially evaluated the causal relationships ranging from 452 metabolites to five infections and detected a total of 71 suggestive associations (*P* < 0.05; corresponding to 64 unique metabolites) by IVW analysis, with 40 associations in 36 known metabolites and 31 associations in 28 unknown metabolites (Supplement Table 1). Among them, 11, 7, 7, 9, and 6 associations were found for known metabolites, and 10, 5, 6, 6, and 4 associations were found for unknown metabolites, respectively related to sepsis, pneumonia, URTI, UTI, and SSTI. Importantly, the minimum F statistic was greater than 10 (ranging from 18.55 to 1431.87), indicating a low likelihood of weak instrument bias (Supplement Table 1). After the multiple-testing correction, we found 4, 2, 3, and 3 associations for known metabolites and 2, 1, 0, and 2 associations for unknown metabolites, respectively significant (FDR < 0.05) for sepsis, pneumonia, URTI, and UTI (Fig. 2). No metabolites significantly associated with SSTI were identified after multiple testing corrections. Specifically, the 7 metabolites associated with sepsis were glycerol (odds ratio [OR] = 1.88, 95% confidence intervals [CIs]: 1.178–2.999, FDR = 0.043), 1-stearoylglycerol (1-SG) (OR = 0.563, 95%CI: 0.374–0.849, FDR = 0.039), 3-carboxy-4-methyl-5-propyl-2-furanpropanoate (CMPF) (OR = 0.806, 95%CI: 0.690–0.942, FDR = 0.033), dihomo-linoleate (20:2n6) (OR = 2.283, 95%CI: 1.334–3.908, FDR = 0.013), X-12407 (OR = 1.212, 95%CI: 1.017–1.445, FDR = 0.047), X-12833 (OR = 1.071, 95%CI: 1.017–1.127, FDR = 0.047), and X-12847 (OR = 1.330, 95%CI: 1.096–1.613, FDR = 0.019).

The three metabolites causally related to pneumonia were ursodeoxycholate (UDCA) (OR = 0.833, 95%CI: 0.726–0.957, FDR = 0.049), kynurenine (OR = 1.685, 95%CI: 1.245–2.282, FDR = 0.004), and X-14588 (OR = 6.202, 95%CI: 1.599–24.051, FDR = 0.042). The three metabolites causally related to URTI are tryptophan (OR = 4.642, 95%CI: 1.709–12.608, FDR = 0.013), histidine (OR = 39.251, 95%CI: 2.640–583.549, FDR = 0.032), and serotonin (5HT) (OR = 0.322, 95%CI: 0.148–0.699, FDR = 0.021).

The five metabolites causally related to UTI are phenylacetate (PA) (OR = 1.476, 95%CI: 1.114–1.956, FDR = 0.033), cysteine (OR = 1.601, 95%CI: 1.207–2.214, FDR = 0.005), eicosenoate (20:1n9 or 11) (OR = 1.544, 95%CI: 1.152–2.070, FDR = 0.018), X-11483 (OR = 1.176, 95%CI: 1.049–1.318, FDR = 0.027), and X-11491 (OR = 1.283, 95%CI: 1.106–1.488, FDR = 0.005).

### Sensitivity analysis

A series of sensitivity analyses were conducted to evaluate the robustness of our main analytical approach. Using the IVW analysis as the basis, we applied MR-Egger regression, weighted median method, and MR-PRESSO to comprehensively evaluate the causal effects between blood metabolites and the infections. The results showed that the analysis results of 12 known metabolites and 5 unknown metabolites were robust. Specifically, the consistent direction and magnitude among the three MR analysis methods are presented in Supplement Table 2 and Supplement Figure 1. After conducting tests for multiple effects and heterogeneity, the *P*-values derived from the Cochran’s Q test and I^2^ indicated no heterogeneity. In addition, we observed a negligible impact of horizontal pleiotropy as evidenced by the small intercept term in the MR-Egger analysis (Table 2). Furthermore, the absence of horizontal pleiotropy or instrumental outliers is supported by MR-PRESSO analysis (P_outlier_ > 0.05). Moreover, the leave-one-out analysis did not reveal any high-influence SNPs that affected the estimate of the combined effect (Supplement Figure 2). Therefore, we identified these 17 metabolites as potential candidate metabolites for further analysis, and specific results are shown in Table 2 and Fig. 2. Furthermore, to confirm the direction of the effect from metabolites to infections, we performed Steiger testing, which revealed that the identified causal relationships were not biased by reverse causation (Supplement Table 2).

**Table 2:** Sensitivity analysis for the causal association between blood metabolites and infection phenotypes

**Metabolite**	**Phenotype**	**Subcategory**	**IVW**		**MR-Egger**		**FDR**_**IVW**_	**correct causal direction**	**steiger_*pval***
			**Q(I**^**2**^**)**	**Q_*pval***	**Intercept**	***P***			
Glycerol	Sepsis	Lipid	17.248 (1.44%)	0.438	0.013	0.172	0.043	TRUE	8.267E-108
1-stearoylglycerol	Sepsis	Lipid	22.401 (0.00%)	0.555	0.014	0.154	0.039	TRUE	3.133E-155
3-carboxy-4-methyl-5-propyl-2-furanpropanoate	Sepsis	Lipid	6.795 (0.00%)	0.871	0.014	0.314	0.033	TRUE	2.379E-98
Dihomo-linoleate (20:2n6)	Sepsis	Fatty acid	7.125 (1.76%)	0.416	0.014	0.520	0.013	TRUE	8.532E-49
X-12407	Sepsis	Unknown metabolite	1.697 (0.00%)	0.989	0.002	0.893	0.047	TRUE	2.597E-93
X-12847	Sepsis	Unknown metabolite	10.864 (17.16%)	0.285	0.009	0.539	0.019	TRUE	3.728E-79
Ursodeoxycholate	Pneumonia	Lipid	9.275 (0.00%)	0.506	0.001	0.890	0.049	TRUE	1.300E-92
Kynurenine	Pneumonia	Amino acid	30.277 (0.00%)	0.651	0.001	0.861	0.004	TRUE	2.497E-263
X-14588	Pneumonia	Unknown metabolite	3.226 (0.00%)	0.919	0.002	0.834	0.042	TRUE	3.242E-69
Tryptophan	URTI	Amino acid	158.647 (5.45%)	0.299	0.018	0.117	0.013	TRUE	0.000E+00
Histidine	URTI	Amino acid	1.301 (0.00%)	0.935	0.003	0.910	0.032	TRUE	3.502E-53
Serotonin (5HT)	URTI	Amino acid	13.247 (0.00%)	0.507	0.009	0.633	0.021	TRUE	5.679E-86
Cysteine	UTI	Amino acid	13.757 (0.00%)	0.684	0.007	0.222	0.005	TRUE	8.899E-120
Phenylacetate	UTI	Amino acid	8.667 (19.23%)	0.277	0.002	0.836	0.033	TRUE	5.894E-58
Eicosenoate (20:1n9 or 11)	UTI	Fatty acid	11.253 (2.25%)	0.422	0.001	0.947	0.018	TRUE	4.033E-76
X-11483	UTI	Unknown metabolite	12.323 (18.85%)	0.264	0.002	0.756	0.027	TRUE	3.474E-122
X-11491	UTI	Unknown metabolite	6.888 (0.00%)	0.908	0.004	0.553	0.005	TRUE	3.973E-129

*Abbreviations*: *IVW* inverse variance weighted, *MR* Mendelian randomization, *FDR* false discovery rate, *URTI* upper respiratory tract infection, *UTI* urinary tract infection, *SSTI* skin and subcutaneous tissue infection

### Confounding analysis

Although sensitivity analyses did not reveal any evidence of bias that would render the MR estimates invalid, we conducted further manual investigations into the second trait (body mass index, body fat percentage, total cholesterol levels, and low-density lipoprotein cholesterol) of the metabolite-associated SNPs. Using Phenoscanner, we removed one SNP (rs3741298) from 1-SG, which was associated with total cholesterol levels, and three SNPs (rs1260326, rs1412972, rs603446) from tryptophan, which were associated with body fat percentage and total cholesterol levels. After performing IVW analysis again, the causal connection ranging from the metabolites to infections remained significant. Specifically, 1-SG (IVW OR = 0.573, 95%CI: 0.380–0.863, FDR = 0.015) and tryptophan (IVW OR = 4.968, 95%CI: 1.789–13.790, FDR = 0.006) were significantly associated with sepsis and URTI, respectively.

### Replication and meta-analysis

**Fig. 3:**
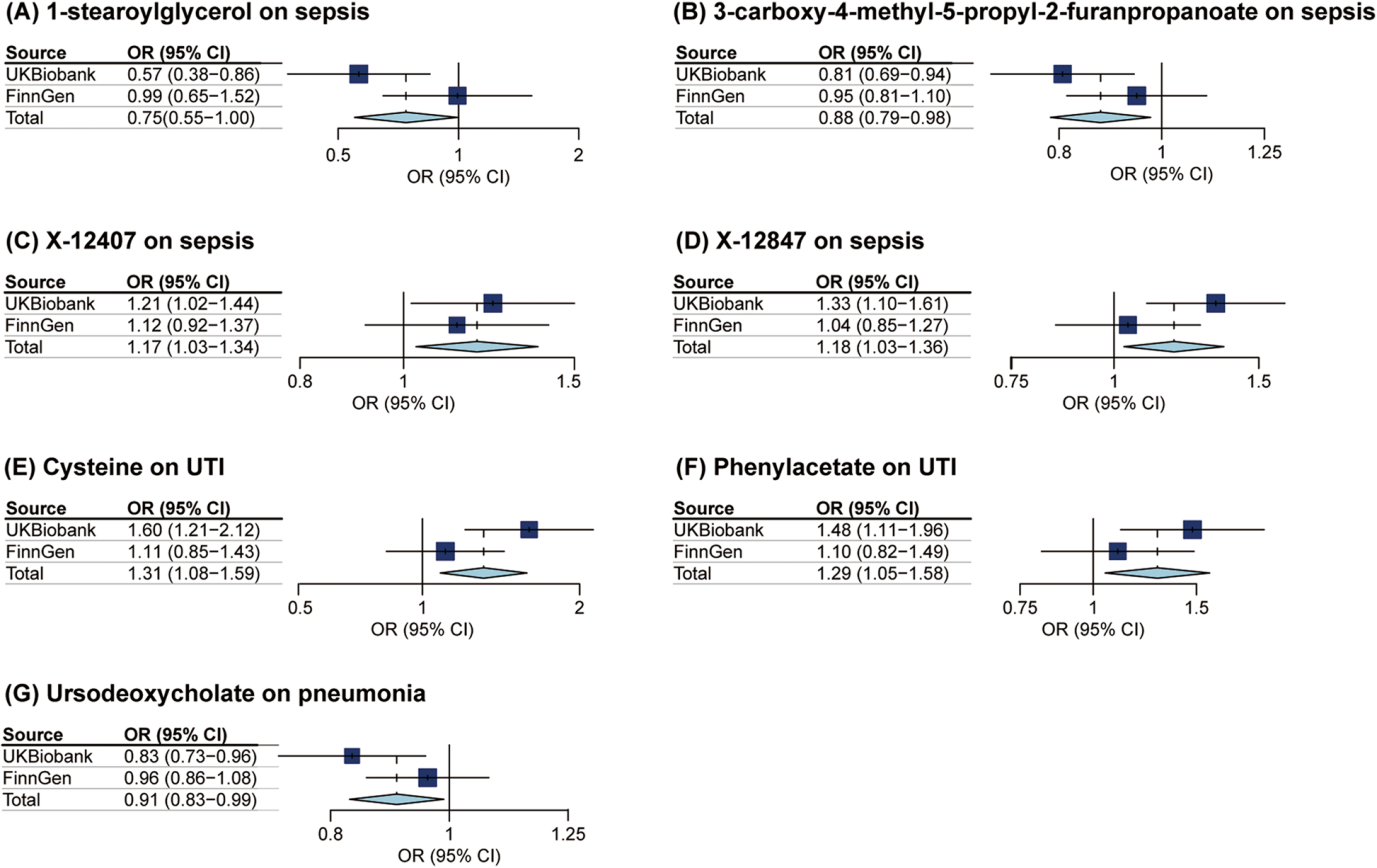
Meta‑analysis of the causal associations between metabolites and 3 types of infection phenotypes (sepsis, pneumonia, and UTI). OR, odds ratio; CI, confidence interval; UTI, urinary tract infection

In order to reinforce the robustness of our findings, we performed replication analyses by utilizing four GWAS datasets from FinnGen R8, which revealed comparable tendencies for some metabolites. with known metabolites, 2, 1, and 2, being respectively linked to the trends of sepsis, pneumonia, and UTI. Additionally, two unidentified metabolites, X-12407 and X-12847, were found to correlate with an elevated risk of sepsis. As shown in Fig. 3, specifically, joint analysis of the UK Biobank and FinnGen datasets further confirmed that high levels of 1-SG (OR = 0.746, 95%CI: 0.573–0.998, *P* = 0.049) and CMPF (OR = 0.875, 95%CI: 0.785–0.976, *P* = 0.017) were protective factors for sepsis, X-12407 (OR = 1.172, 95%CI:1.028–1.336, *P* = 0.018) and X-12847 (OR = 1.183, 95%CI: 1.028–1.360, *P* = 0.019) are risk factors for sepsis. UDCA (OR = 0.906, 95%CI: 0.829–0.990, *P* = 0.029) was a protective factor for pneumonia. High levels of PA (OR = 1.287, 95%CI: 1.048–1.579, *P* = 0.016) and cysteine (OR = 1.310, 95%CI: 1.082–1.586, *P* = 0.006) predicted a higher risk of UTI.

We observed null estimates in tryptophan, serotonin (5HT), dihomo-linoleate (20:2n6), glycerol, kynurenine, histidine, eicosenoate (20:1n9 or 11), X-14588, X-11483, and X-11491 in the meta-analysis. Moreover, replication analyses using the GWAS summary data from FinnGen database revealed divergent directions. Details can be found in Supplement Figure 3.

### Multivariable and reverse MR Analysis

Additionally, the Meta-analysis findings suggest that several metabolites may affect both sepsis and pneumonia. To explore the unique effects of each metabolite on sepsis or pneumonia, we performed a multivariable MR analysis. Interestingly, we found that the causal effect of each metabolite was consistent in direction and magnitude with the unadjusted results obtained through the IVW method (Table 3). Sepcifically the four metabolites that had independent causal effects on sepsis were 1-SG (OR = 0.561, 95%CI: 0.403–0.780, *P* < 0.001), CMPF (OR = 0.780, 95%CI: 0.6899–0.883, *P* < 0.001), X-12407 (OR = 1.294, 95%CI: 1.131–1.481, *P* < 0.001), and X-12847 (OR = 1.344, 95%CI: 1.152–1.568, *P* < 0.001). In addition, significant causal effects were observed for PA (OR = 1.426, 95%CI: 1.152–1.765, *P* = 0.001) and cysteine (OR = 1.522, 95%CI: 1.170–1.980, *P* = 0.002) on UTI.

**Table 3:** Estimated causal effects of metabolites on sepsis/UTI by the multivariable Mendelian randomization analysis

Metabolite	Infections	Nsnp	Multivariable MR	
			**OR (95% CI)**	***P***
1-stearoylglycerol	Sepsis	228	0.561 (0.403–0.780)	5.859E-04
3-carboxy-4-methyl-5-propyl-2-furanpropanoate	Sepsis	228	0.780 (0.689–0.883)	9.070E-05
X-12407	Sepsis	228	1.294 (1.131–1.481)	1.745E-04
X-12847	Sepsis	228	1.344 (1.152–1.568)	1.706E-04
Phenylacetate	UTI	54	1.426 (1.152–1.765)	1.098E-03
Cysteine	UTI	54	1.522 (1.170–1.980)	1.743E-03

*Abbreviations*: *MR* Mendelian randomization, *OR* odds ratio, *CI* confidence interval, *UTI* urinary tract infection

Finally, to further investigate the causality between metabolites and infection phenotypes, we conducted a reverse MR analysis using instrumental variables that represent sepsis, pneumonia, and UTI, respectively. By selecting top independent SNPs with a significance level of *P* < 1 × 10–5 as instrumental variables and performing MR estimation, we sought to determine if there was any evidence of a reverse causal correlation from the 7 identified metabolites to the four infections. However, our analysis revealed limited support for such a relationship, as demonstrated by Supplement Table 3.

## Discussion

In this study, we conducted a comprehensive two-sample MR analysis using GWAS summary statistics to assess potential associations between 452 metabolites and five types of infection phenotypes (sepsis, pneumonia, URTI, UTI, and SSTI). The inferred causal relationships were robust. Our findings revealed 7 metabolites with statistically significant associations, even after multiple testing corrections, including 2 previously unknown metabolites. We eliminated the potential for reverse causation and validated that the identified metabolites are precursors, rather than consequences, of infection phenotypes. Specifically, a genetically determined higher level of 1-SG and CMPF in the blood is causally linked to a lower risk of sepsis, while higher levels of phenylacetate and cysteine predict a higher risk of UTI, and UDCA is a protective factor for pneumonia. We did not identify any blood metabolites with clear associations with URTI and SSTI. This information has practical implications for healthcare providers who can use it to identify at-risk patients and intervene early to prevent or treat these infections. By understanding which metabolites are involved in the development and progression of these diseases, healthcare providers can develop effective screening and prevention strategies for these infectious diseases in clinical practice. As far as we are aware, this was the first systematic evaluation of the causal effects of human metabolites on five infections using MR analysis.

Sepsis is a disease caused by infections that can lead to organ dysfunction and death. It is one of the leading causes of mortality worldwide, with nearly 6 million people dying from sepsis annually [24]. Early diagnosis and treatment of sepsis are crucial for patients who may be at risk. However, traditional observational studies are challenged by small sample sizes and confounding factors, making the early prediction of sepsis outcomes difficult. By using MR studies with little reverse causality and confounding, we found that there was a causal connection from high levels of blood 1-SG and CMPF to a lower risk of sepsis among known metabolites. Additionally, genetic susceptibility to sepsis development was found to be promoted by increased levels of unknown metabolites X-12407 and X-12847. However, there have been few studies investigating the role of 1-stearoylglycerol and CMPF in sepsis. 1-SG is a lipid metabolite that is primarily converted to free fatty acids for further metabolism by monoacylglycerol lipase (MAGL). Elevated levels of MAGL are usually positively correlated with the body’s inflammatory state. Studies have shown that inhibiting MAGL with inhibitors can exert anti-inflammatory properties and protective effects in experimental models of neuroinflammation [25] and traumatic brain injury [26]. In addition, compared to the control group, the MAGL inhibition group significantly reduced the production of the pro-inflammatory cytokine IL-6 and increases the production of the anti-inflammatory cytokine IL-10 [27]. These finding suggests that 1-SG may have exert anti-inflammatory properties to prevent sepsis occurrence through MAGL. However, these results were derived from experimental models and further research is needed to determine the potential roles of 1-SG and MAGL inhibitors in the treatment of human diseases. CMPF, a major furan fatty acid metabolite, has been controversial in its role in disease. Some studies have identified CMPF as a uremic toxin [28] that increases reactive oxygen species production and induces renal injury in human kidney cells [29]. However, other studies have suggested that higher CMPF levels are associated with reduced risk of all-cause mortality [30] and periodontitis [31]. These equivocal findings may be due to methodological defects such as residual confounding. By utilizing the MR study without reverse causality and confounding, we provide causal genetic evidence that CMPF has a protective effect on sepsis, offering new insights into the role of CMPF in the field of infection and providing theoretical support for further research on sepsis.

Furthermore, two other unknown metabolites, X-12407 and X-12847, were also confirmed to be causally correlated with sepsis. However, due to their elusive structural and functional characteristics, extracting further interesting insights from them proves challenging. Nevertheless, our MR study provides new avenues for exploring these enigmatic metabolites, and their successful identification promises to greatly facilitate the discovery of biomarkers and the study of infectious diseases.

Pneumonia has always been acknowledged as a formidable disease. Data from the Organization for Economic Co-operation and Development (OECD) countries reveals that despite enhanced healthcare access and sophisticated life support systems, including the utilization of extracorporeal membrane oxygenation (ECMO), pneumonia still contributes to 30% of all respiratory deaths [32]. Therefore, finding some modifiable factors for early diagnosis and treatment of pneumonia may be crucial for patients with pneumonia. Our MR study has identified UDCA as a protective factor against Pneumonia, which may have important practical implications. UDCA is a secondary bile acid commonly used for the treatment of chronic hepatitis, cholestatic liver disease [33], and primary biliary cholangitis [34]. Recent research has also suggested that UDCA can downregulate angiotensin converting enzyme 2 (ACE) in human lung, intestinal, and biliary organoids in vitro [35], potentially preventing SARS-CoV-2 infection and improving clinical outcomes following COVID-19 infection [35]. These findings suggest that UDCA may have the potential in preventing infections and reducing the severity of COVID-19, consistent with our research findings. An animal experiment may explain the mechanism behind this, as UDCA has been shown to suppress the production of pro-inflammatory cytokines such as TNF-α, IL-1β, IL-2, IL-4, and IL-6 at the mRNA and protein levels [36]. However, the specific biological pathways linking UDCA to the pathophysiology of Pneumonia are yet to be fully illuminated. Further investigation is necessary to confirm its potential mechanisms of action, and the precise mechanisms will require extensive clinical trials to establish.

Our study also revealed that elevated levels of two metabolites, Cysteine and Phenylacetate (PA), have detrimental effects on the occurrence of UTI. While the majority of UTIs are usually not severe, neglecting proper care can give rise to critical complications like acute renal failure and sepsis [37]. Hence, the early identification of risk factors for the occurrence of UTI and intervention becomes particularly crucial.

Although no previous research had explored their association with UTI, some observational studies and cellular experiments partially support the unfavorable role of cysteine in disease onset. Cysteine, a semi-essential amino acid and a precursor to biothiols homocysteine and glutathione, has been observed to cause neurotoxicity, adverse pregnancy outcomes, and rheumatoid arthritis in observational studies [38]. In vitro experiments have also revealed that cysteine promotes survival and invasiveness of ovarian cancer cells, leading to poorer disease prognosis [39]. On the other hand, PA, a common metabolite of phenylalanine, is widely present in organisms and controls growth and differentiation [and controls growth and differentiation in a wide spectrum of organisms, but its relationship with UTI has rarely been studied. Previous observational studies have shown that elevated levels of Phenylacetate are often associated with overgrowth of urine microbiota, indicating urinary dysbiosis [40]. Additionally, some studies have found that PA synergizes with bird’s amino acid to treat hyperammonemia associated with urea cycle disorders [41], promoting the excretion of waste nitrogen. These ambiguous results make it difficult to draw any causal inference since the association may be confounded by various factors. In contrast, MR studies inherently possess the advantage of excluding confounding factors. Our MR analysis indeed suggests a causal role of PA and cysteine in UTI onset, indicating that they could be promising therapeutic targets and providing valuable clues for novel UTI treatments. However, further clinical research is needed to validate these findings.

There are several positive aspects of our investigation, including the usage of a Mendelian randomization design, integration of data from different sources, and bidirectional MR analysis to explore the causal relationship between the exposure and risk of infections, which has strong clinical implications. Moreover, our study employed rigorous quality assurance standards and multiple analytical approaches to ensure the reliability of the MR analysis. However, there are some limitations to our study. Firstly, due to the limited number of SNPs reaching genome-wide significance, we relaxed the threshold, although each SNP’s F-statistic was greater than 10, indicating the exclusion of weak instrumental variables, there may still be some bias. Secondly, the data are derived from European populations, this may hamper the applicability of our findings to a broader population, although it also has the advantage of reducing population structure bias. Lastly, the conclusion drawn from this Mendelian study has yet to be validated by molecular biology or biochemistry experiments, so further randomized controlled trials with a larger population are needed to confirm the causal relationship.

## Conclusions

This study has uncovered causal relationships from blood metabolites to various infections. Specifically, the elevation of 1-SG, CMPF, and UDCA plays a causal impact in reducing the risk of sepsis and pneumonia, while the elevation of PA and cysteine has a causal relationship with the occurrence of UTI. This discovery may have profound implications for understanding these diseases’ etiology and clinical prevention and treatment.

### Supplementary Information

**Additional file 1: Supplement Figure 1.** Scatterplot for the significant Mendelian randomization (MR) association (FDR < 0.05) between metabolites and 4 types of infection phenotypes (sepsis, pneumonia, URTI, and UTI). SNP, single nucleotide polymorphism; URTI, upper respiratory tract infection; UTI, urinary tract infection. **Supplement Figure 2.** Forest plots for the Mendelian randomization (MR) leave-one-out analysis of the significant inverse variance weighted (IVW) estimates. URTI, upper respiratory tract infection; UTI, urinary tract infection. **Supplement Figure 3.** Meta‑analysis of the causal associations between metabolites and 4 types of infection phenotypes (sepsis, pneumonia, URTI, and UTI). OR, odds ratio; CI, confidence interval; URTI, upper respiratory tract infection; UTI, urinary tract infection.

**Additional file 2: Table S1.** Inverse-variance weighted Mendelian randomization estamites for metabolites on infection phenotypes (*P*<0.05). **Table S2.** Three different Mendelian randomization methods to evaluate the effects of metabolites on infection phenotypes. **Table S3.** Reverse associations for sepsis/UTI/pneumonia on four metabolites.

## Data Availability

The datasets analyzed in this study are publicly available summary statistics. The human blood metabolites datasets were publicly available from Metabolomics GWAS Server at http://metabolomics.helmholtz-muenchen.de/gwas/. GWAS summary statistics of sepsis, pneumonia, URTI, and UTI were publicly available from UK Biobank (https://www.ukbiobank.ac.uk/datasets/) and FinnGen consortium (https://www.finngen.fi/en/access_results). We used the following web-based resources: Phenoscanner (http://www.phenoscanner.medschl.cam.ac.uk/), and IEU OpenGWAS (https://gwas.mrcieu.ac.uk/).

## Abbreviations

GWAS: Genome-wide association study
MR: Mendelian randomization
MR-PRESSO: Mendelian Randomization Pleiotropy RESidual Sum and Outlier
OR: Odds ratio
CI: Confidence interval
FDR: False discovery rate
SNPs: Single-nucleotide polymorphisms
RCT: Randomized controlled trial
IV: Instrumental variable
URTI: Upper respiratory tract infection
UTI: Urinary tract infection
SSTI: Skin and subcutaneous tissue infection
1-SG: 1-Stearoylglycerol
CMPF: 3-Carboxy-4-methyl-5-propyl-2-furanpropanoate
PA: Phenylacetate
UDCA: Ursodeoxycholate
KEGG: Kyoto Encyclopedia of Genes and Genomes
EAF: Effect allele frequency
IVW: Inverse variance weighted
WM: Weighted median
LOO: Leave-one-out
MAGL: Monoacylglycerol lipase
