# Supplementary material for: Causal relationship between blood metabolites and risk of five infections: a Mendelian randomization study
Source: BMC Infect Dis. 2023 Oct 7;23:663. doi: 10.1186/s12879-023-08662-6 (PMC10559484; doi:10.1186/s12879-023-08662-6)
Supplement: Supplementary file 1 — Additional file 1: Supplement Figure 1. Scatterplot for the significant Mendelian randomization (MR) association (FDR < 0.05) between metabolites and 4 types of infection phenotypes (sepsis, pneumonia, URTI, and UTI). SNP, single nucleotide polymorphism; URTI, upper respiratory tract infection; UTI, urinary tract infection. Supplement Figure 2. Forest plots for the Mendelian randomization (MR) leave-one-out analysis of the significant inverse variance weighted (IVW) estimates. URTI, upper respiratory tract infection; UTI, urinary tract infection. Supplement Figure 3. Meta‑analysis of the causal associations between metabolites and 4 types of infection phenotypes (sepsis, pneumonia, URTI, and UTI). OR, odds ratio; CI, confidence interval; URTI, upper respiratory tract infection; UTI, urinary tract infection. [file 12879_2023_8662_MOESM1_ESM.docx]

**Supplementary Material**

**Supplement Figure-1:** Scatterplot for the significant Mendelian randomization (MR) association (FDR < 0.05) between metabolites and 4 types of infection phenotypes (sepsis, pneumonia, URTI, and UTI). SNP, single nucleotide polymorphism; URTI, upper respiratory tract infection; UTI, urinary tract infection.

Within each panel, the black points represent the causal estimate of the association between a specific SNP and one of the infection phenotypes (sepsis, pneumonia, URTI, and UTI). Each scatter point represents the MR estimation results of a SNP-disease pair. The lines in the plot indicate the overall estimates of the causal effect between the metabolite and the disease using different MR methods.

**Supplement Figure-2：** Forest plots for the Mendelian randomization (MR) leave-one-out analysis of the significant inverse variance weighted (IVW) estimates. URTI, upper respiratory tract infection; UTI, urinary tract infection.

Within each panel, the black points represent the causal estimate of the association between a specific metabolite and 4 types of infection phenotypes (sepsis, pneumonia, URTI, and UTI) after discarding each SNP in turn. Red points represent the pooled IVW estimates. Horizontal lines denote 95% confidence intervals.

**Supplement Figure-3:** Meta‑analysis of the causal associations between metabolites and 4 types of infection phenotypes (sepsis, pneumonia, URTI, and UTI). OR, odds ratio; CI, confidence interval; URTI, upper respiratory tract infection; UTI, urinary tract infection.


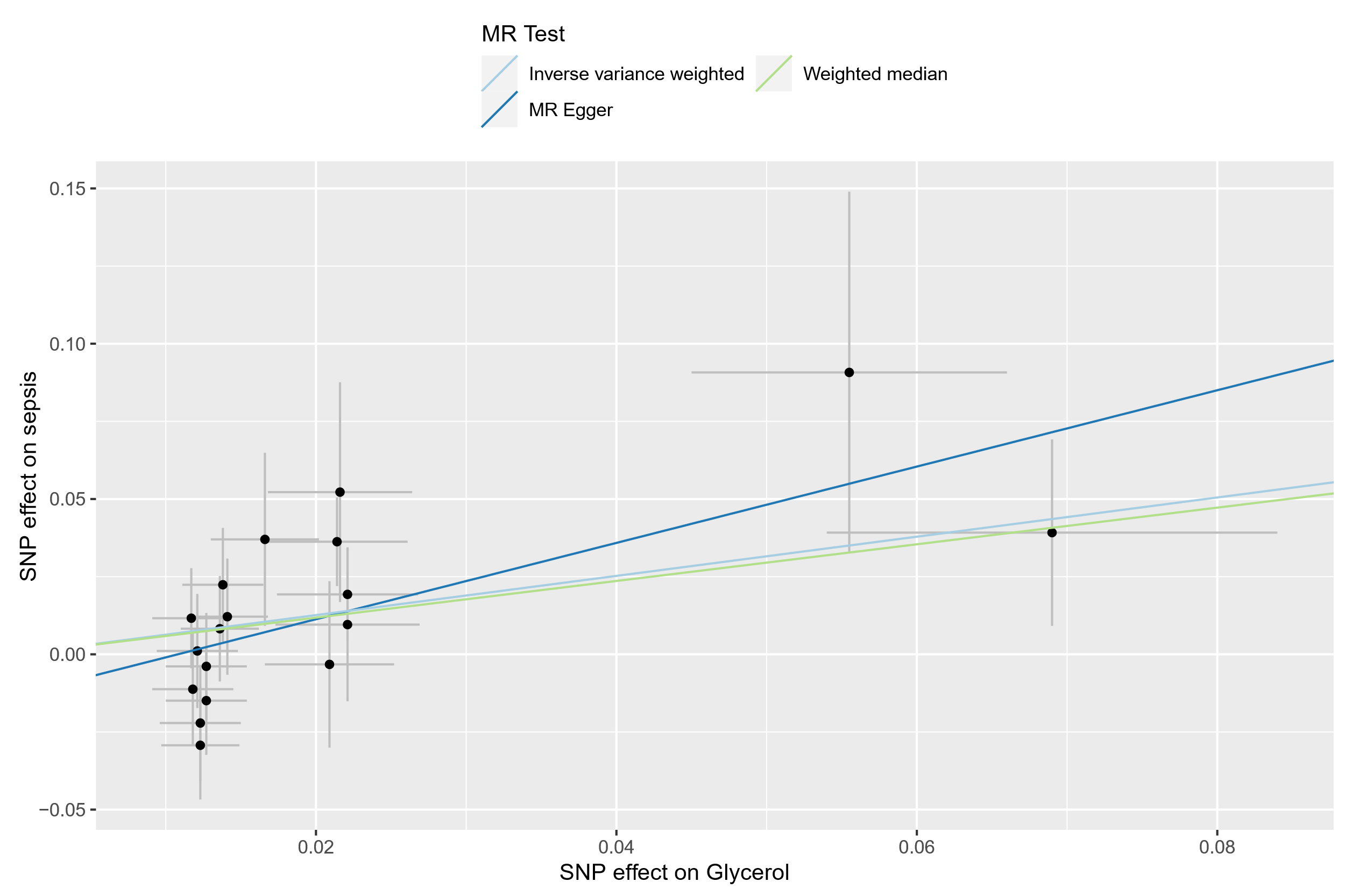

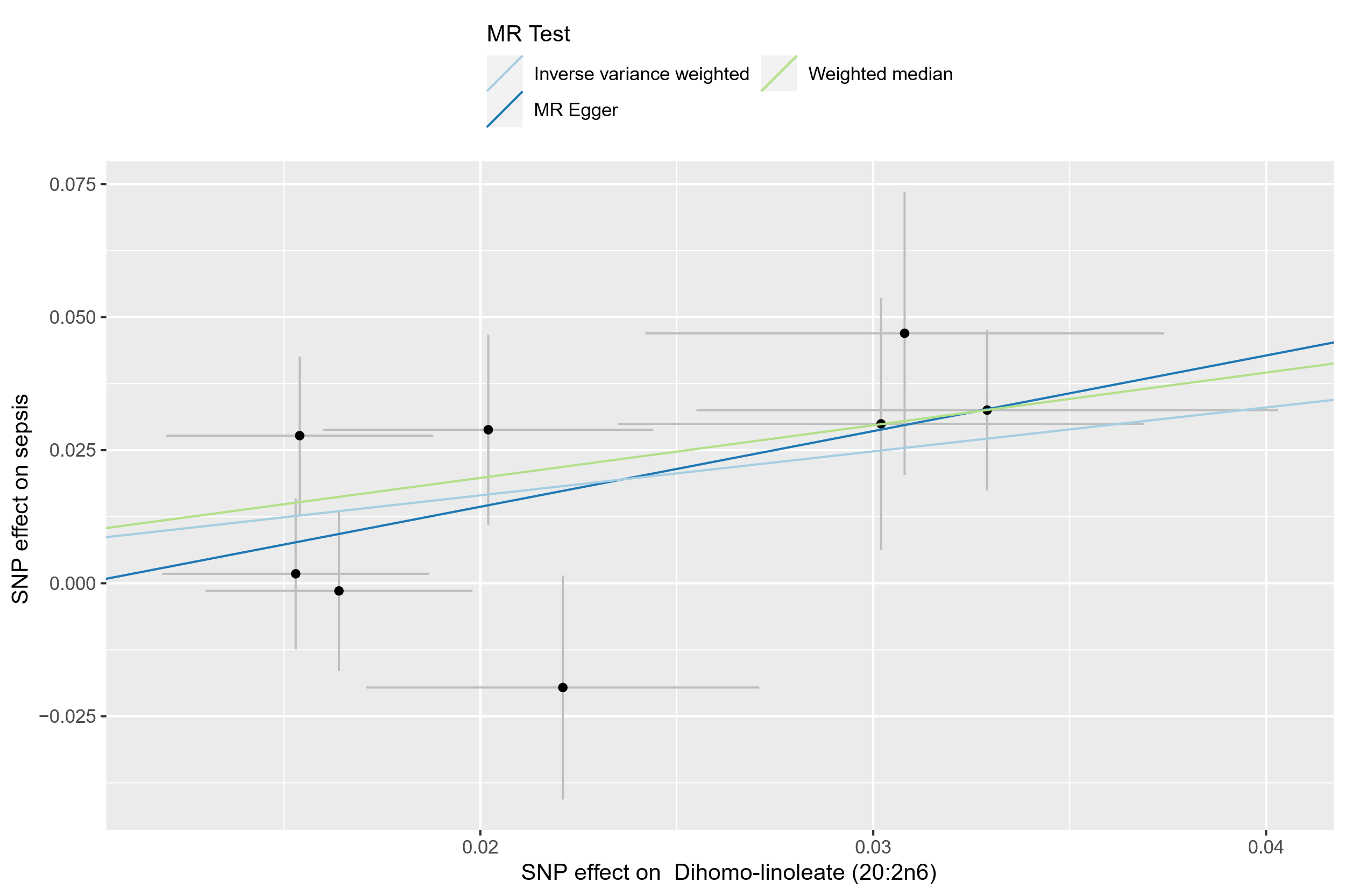

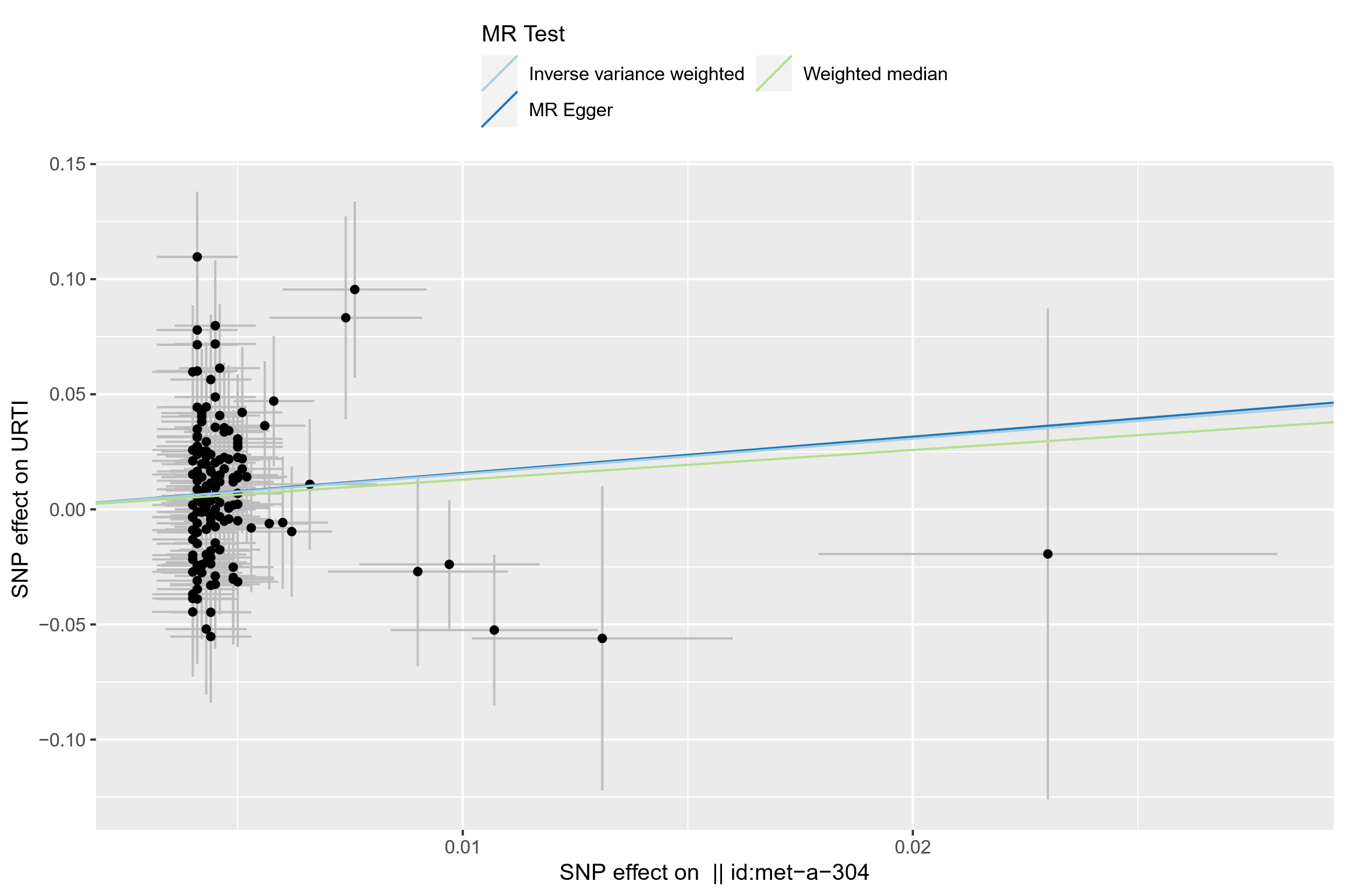

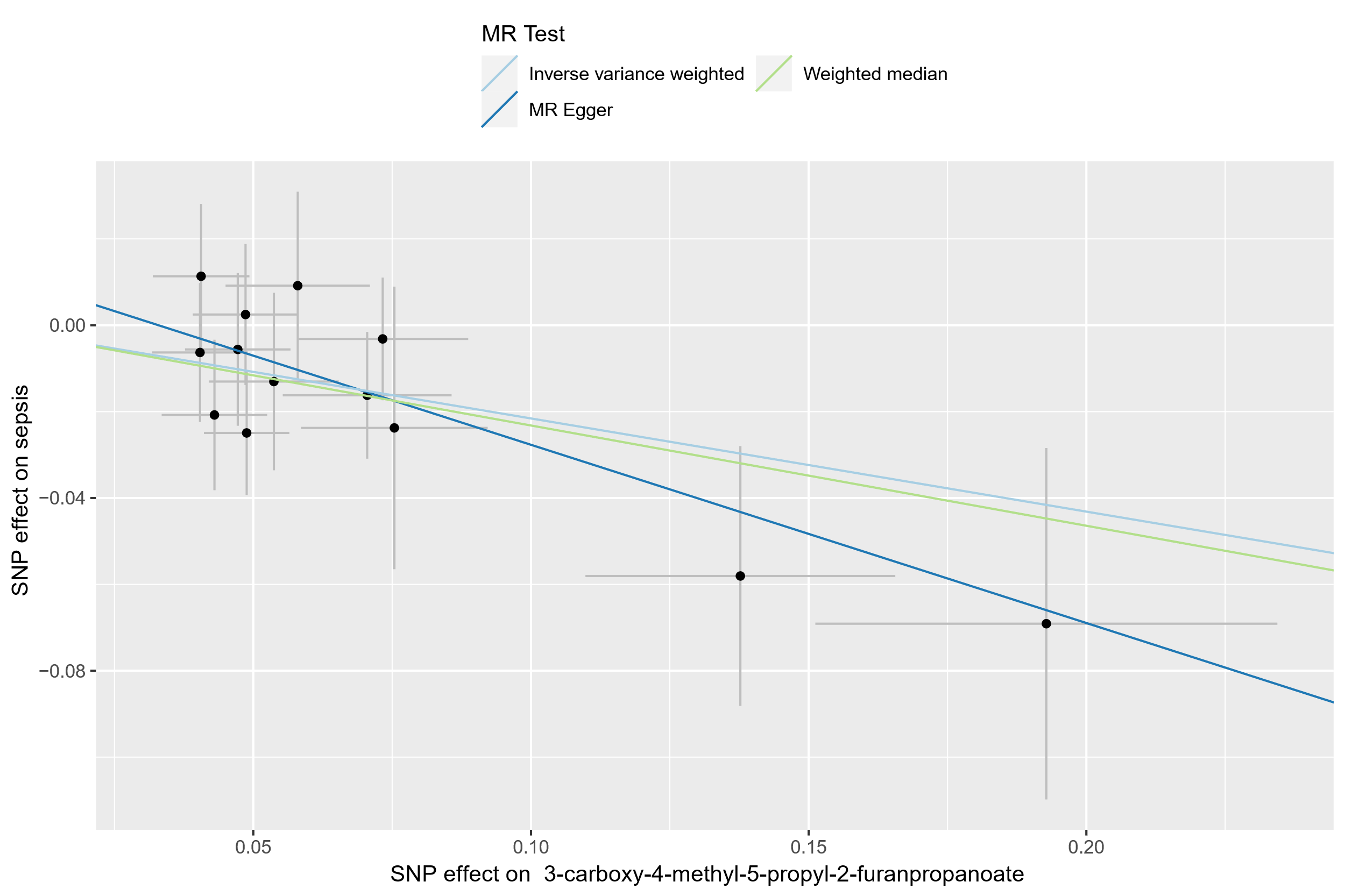

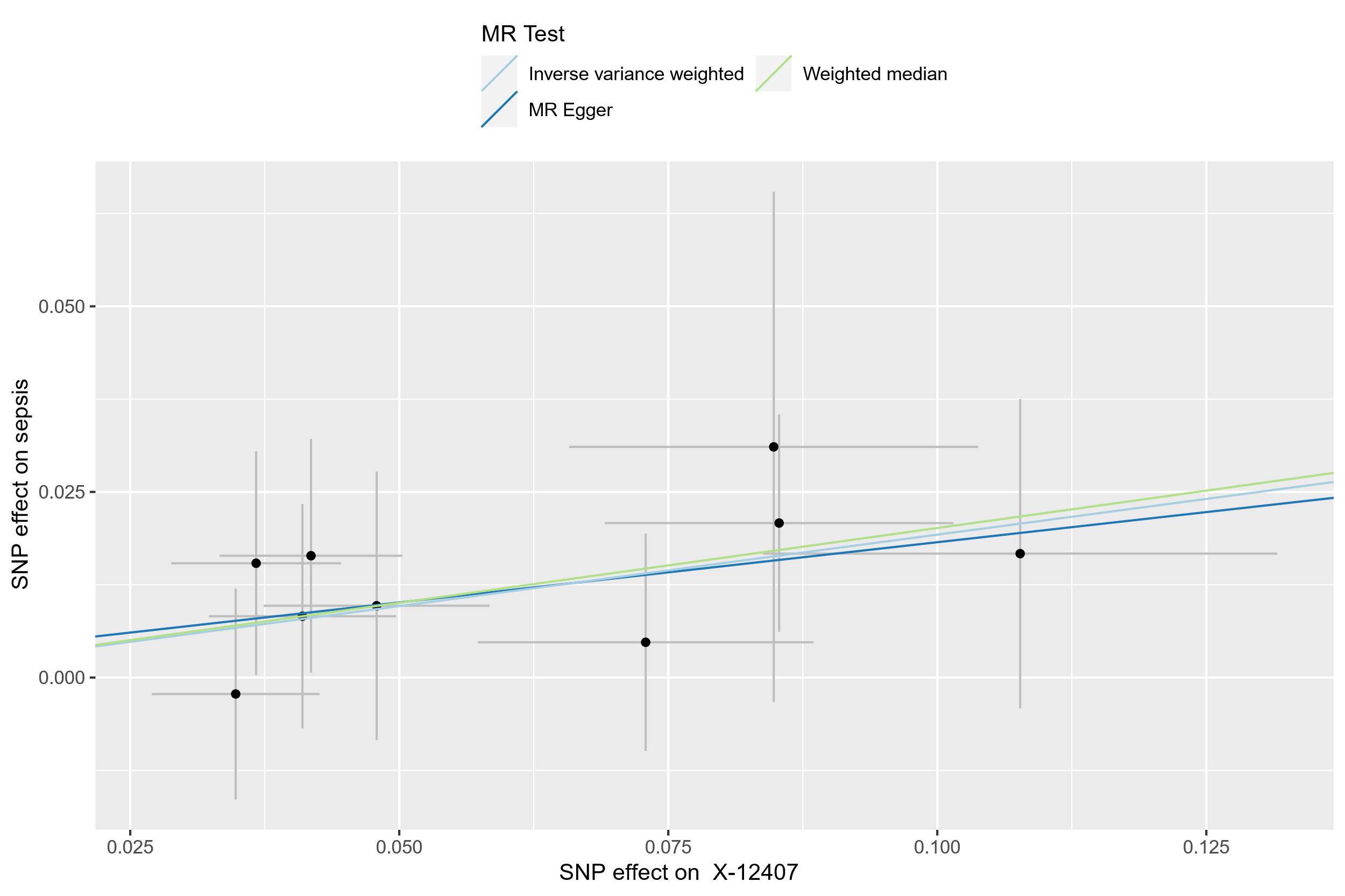

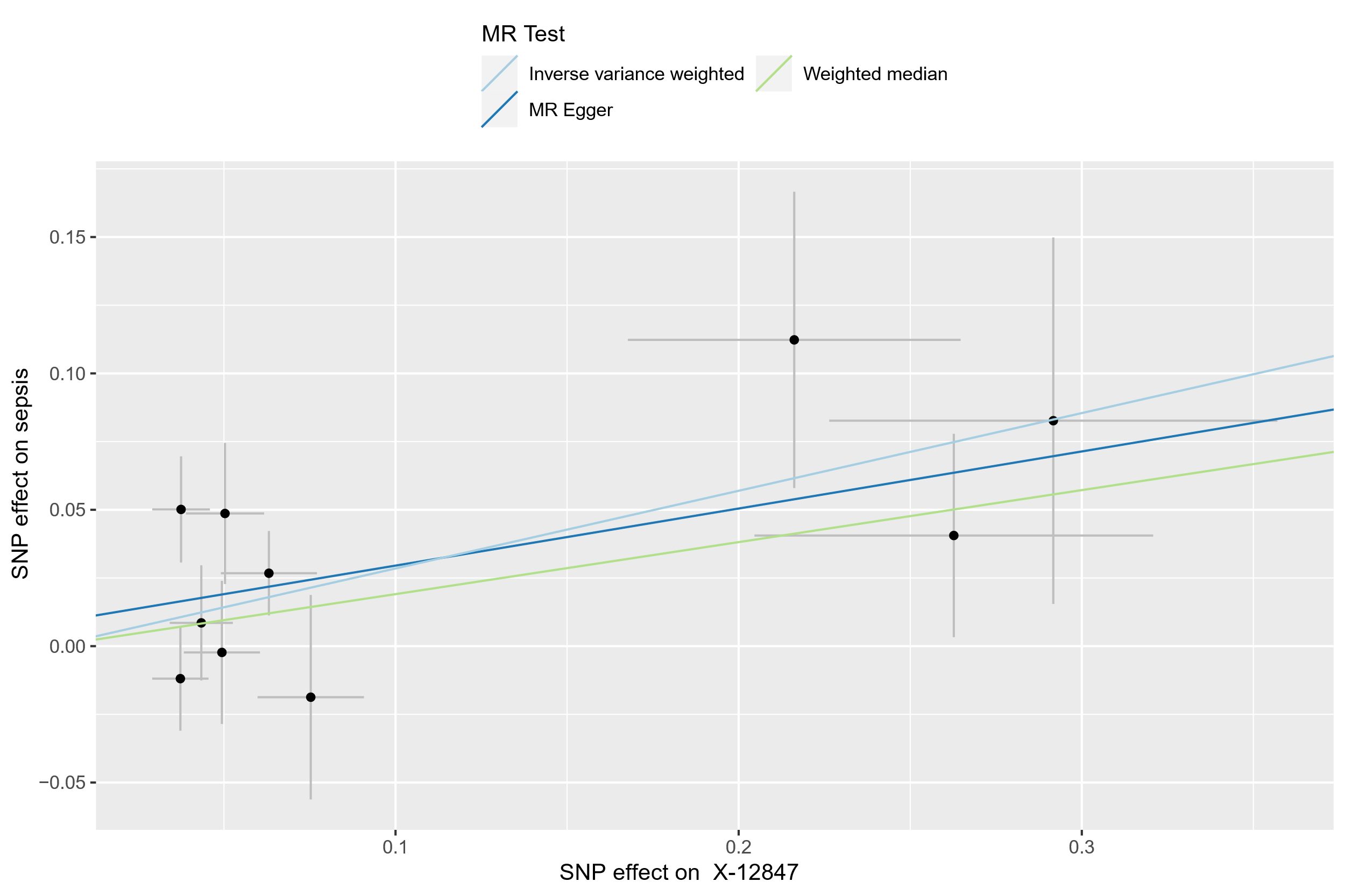

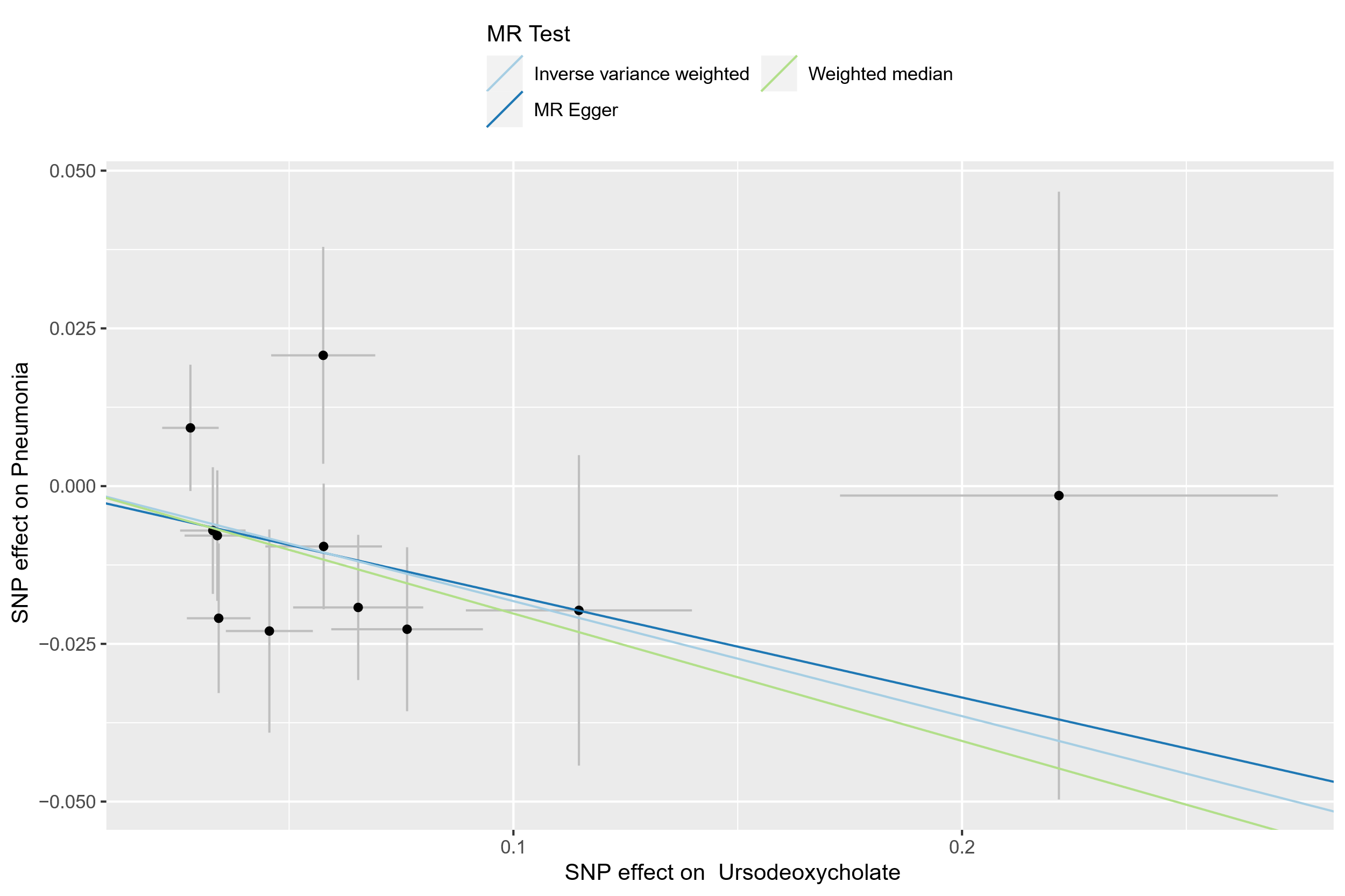

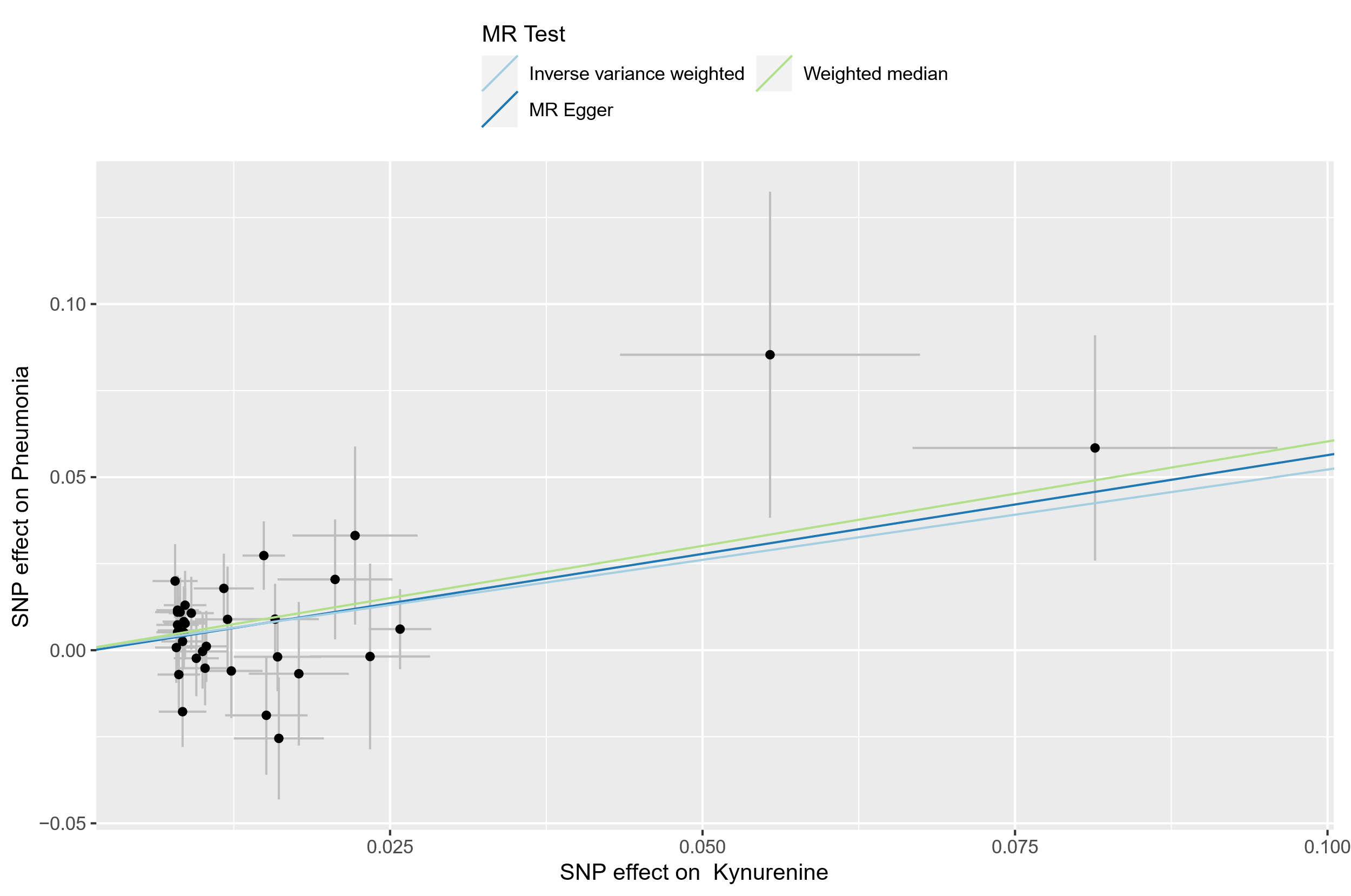

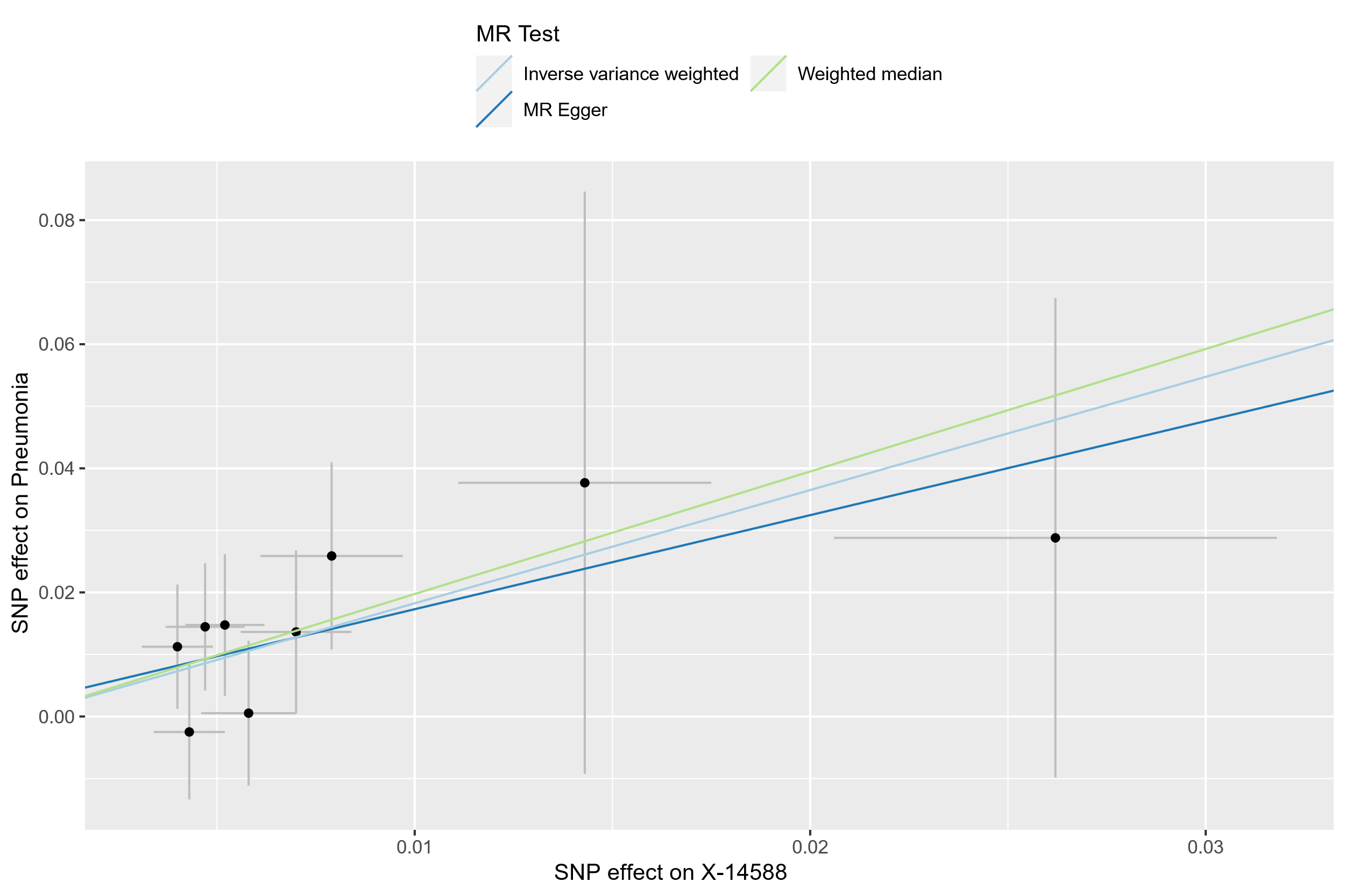

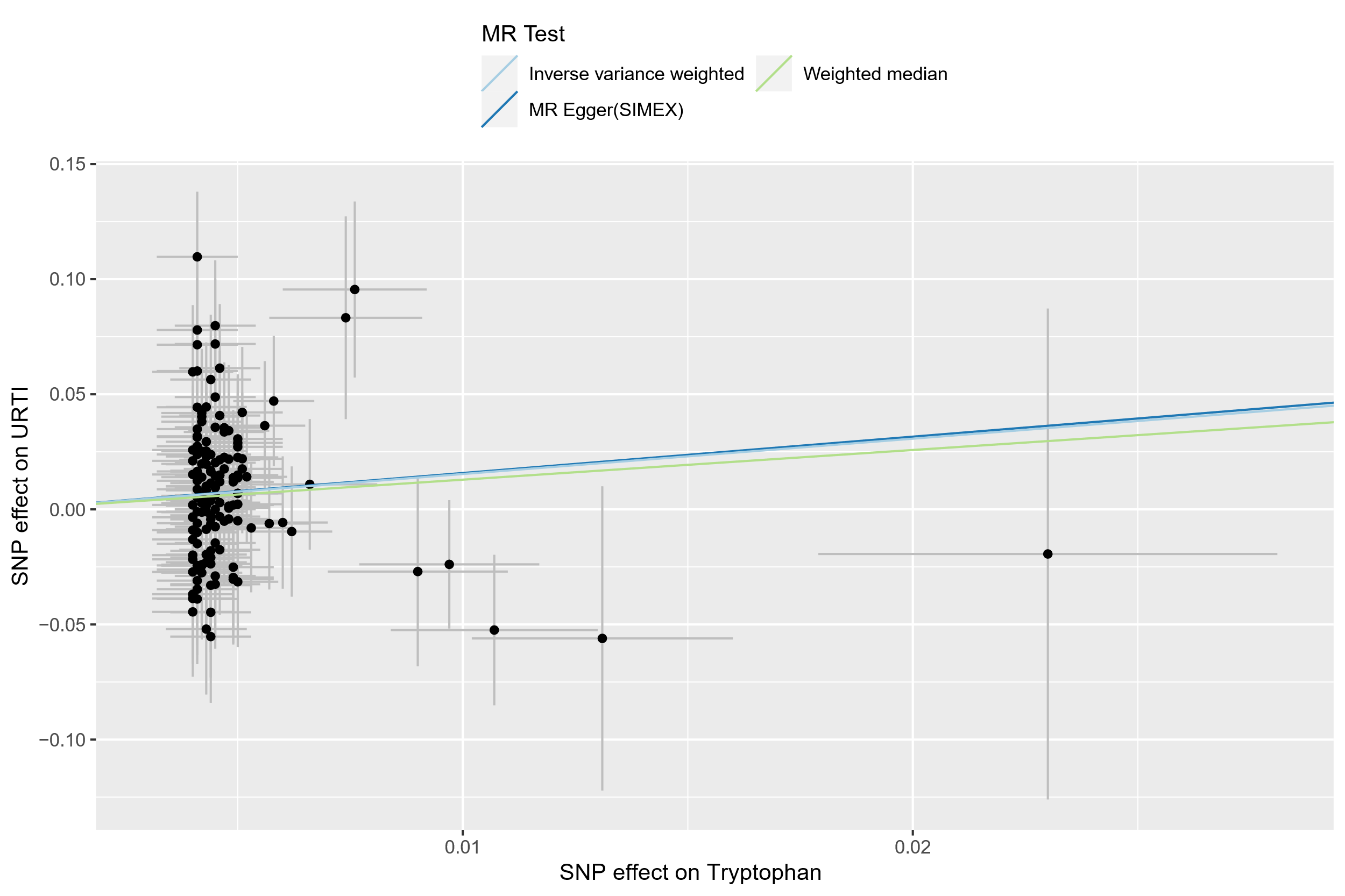

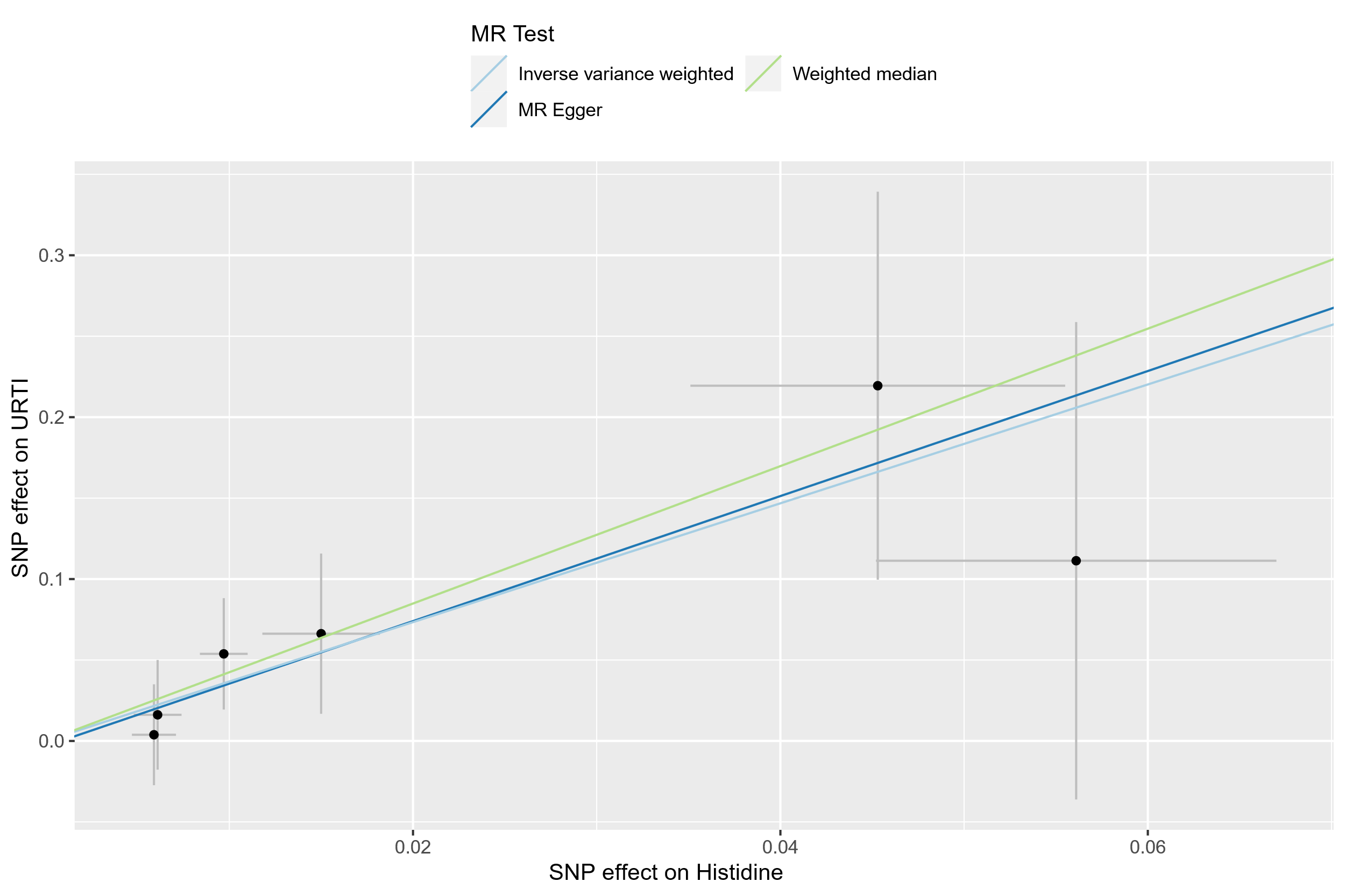

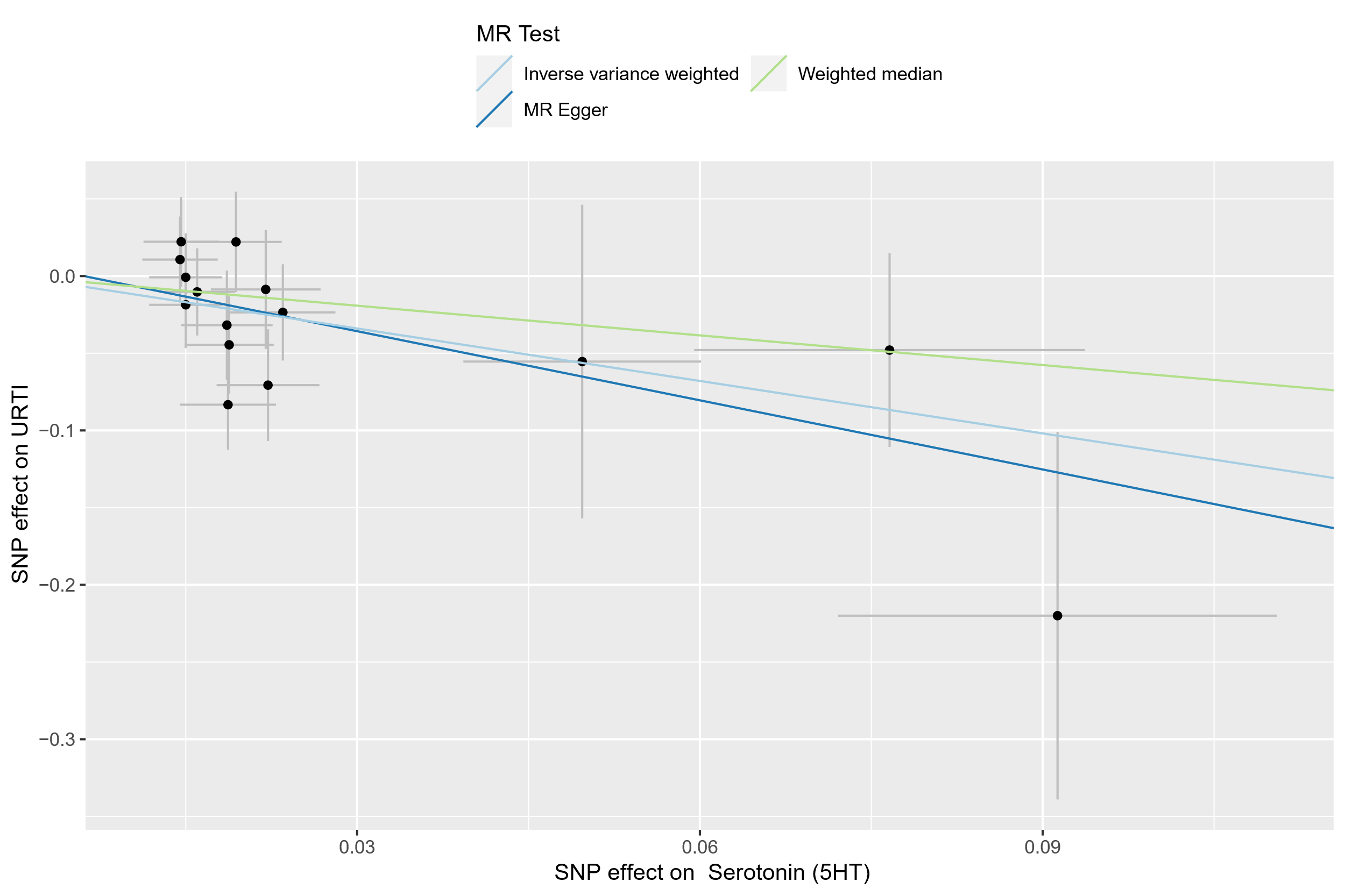

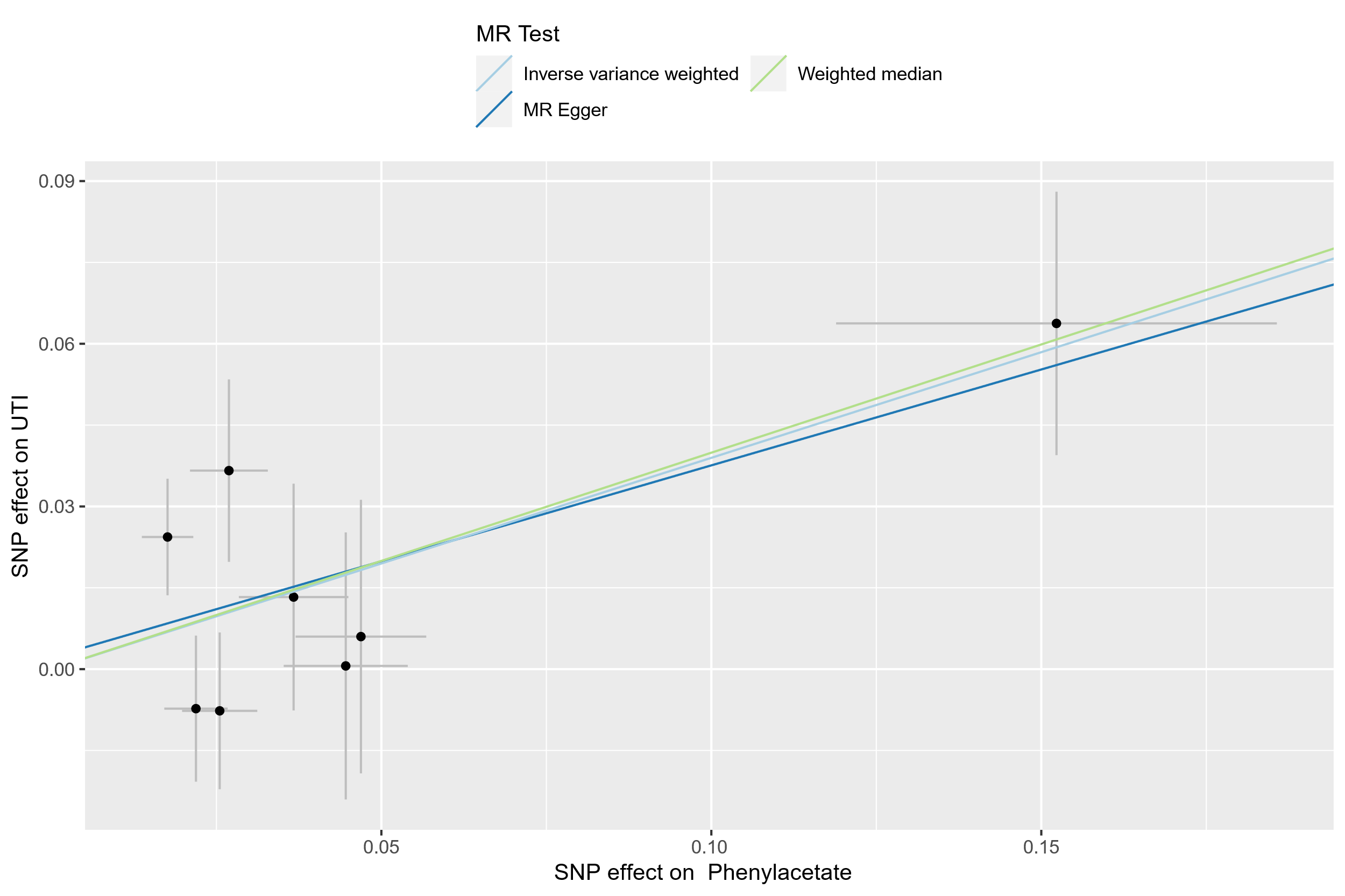

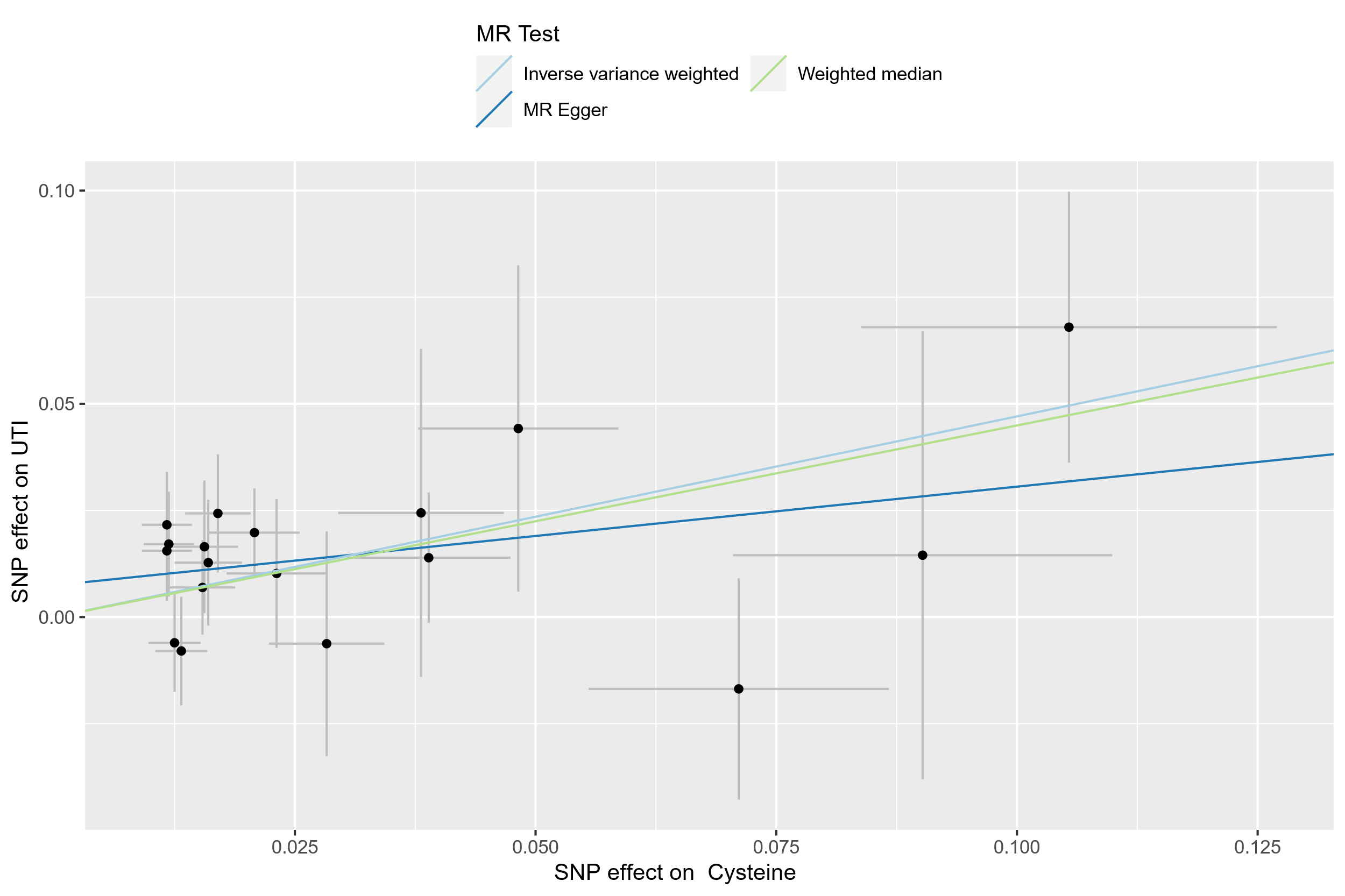

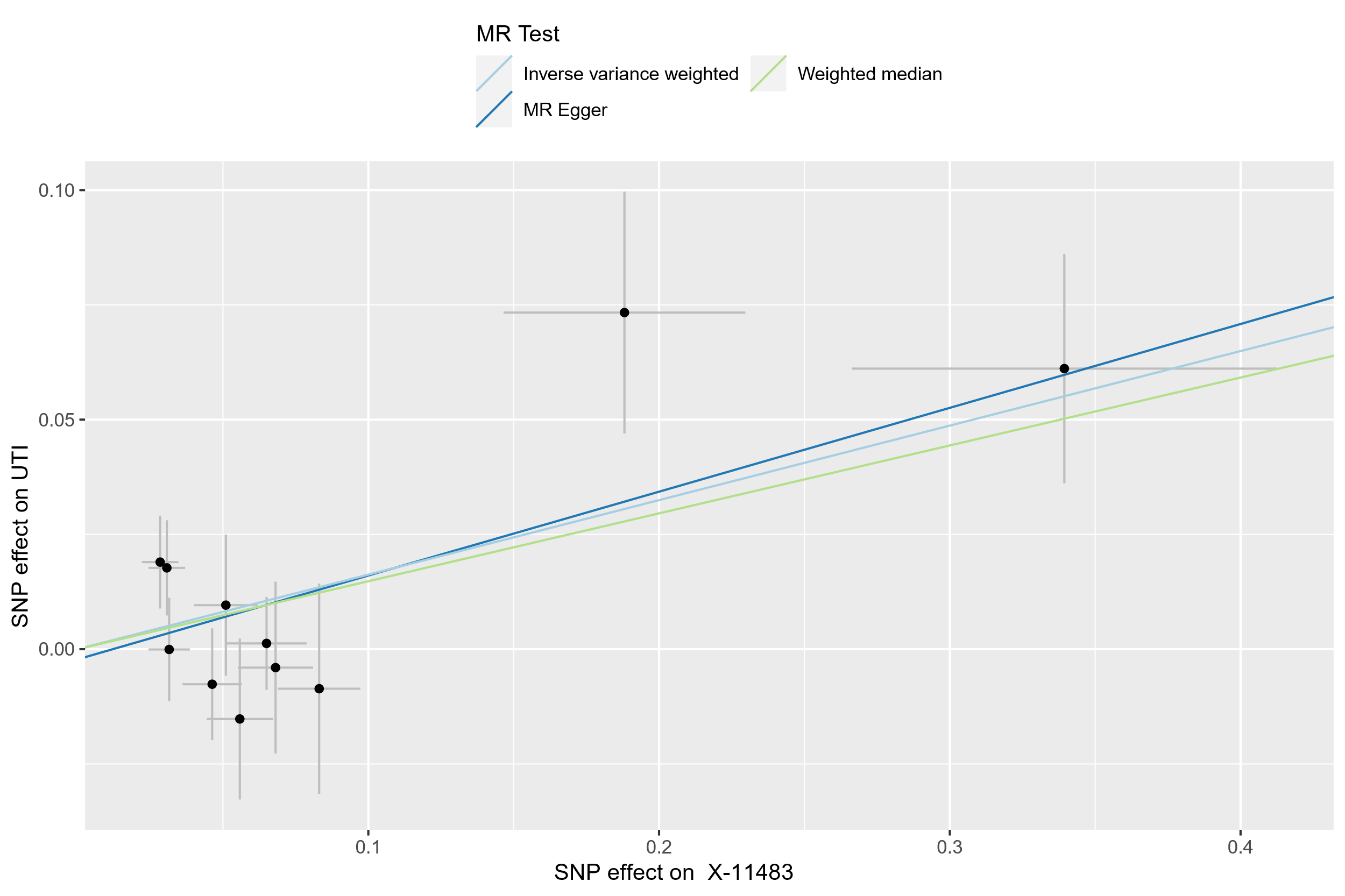

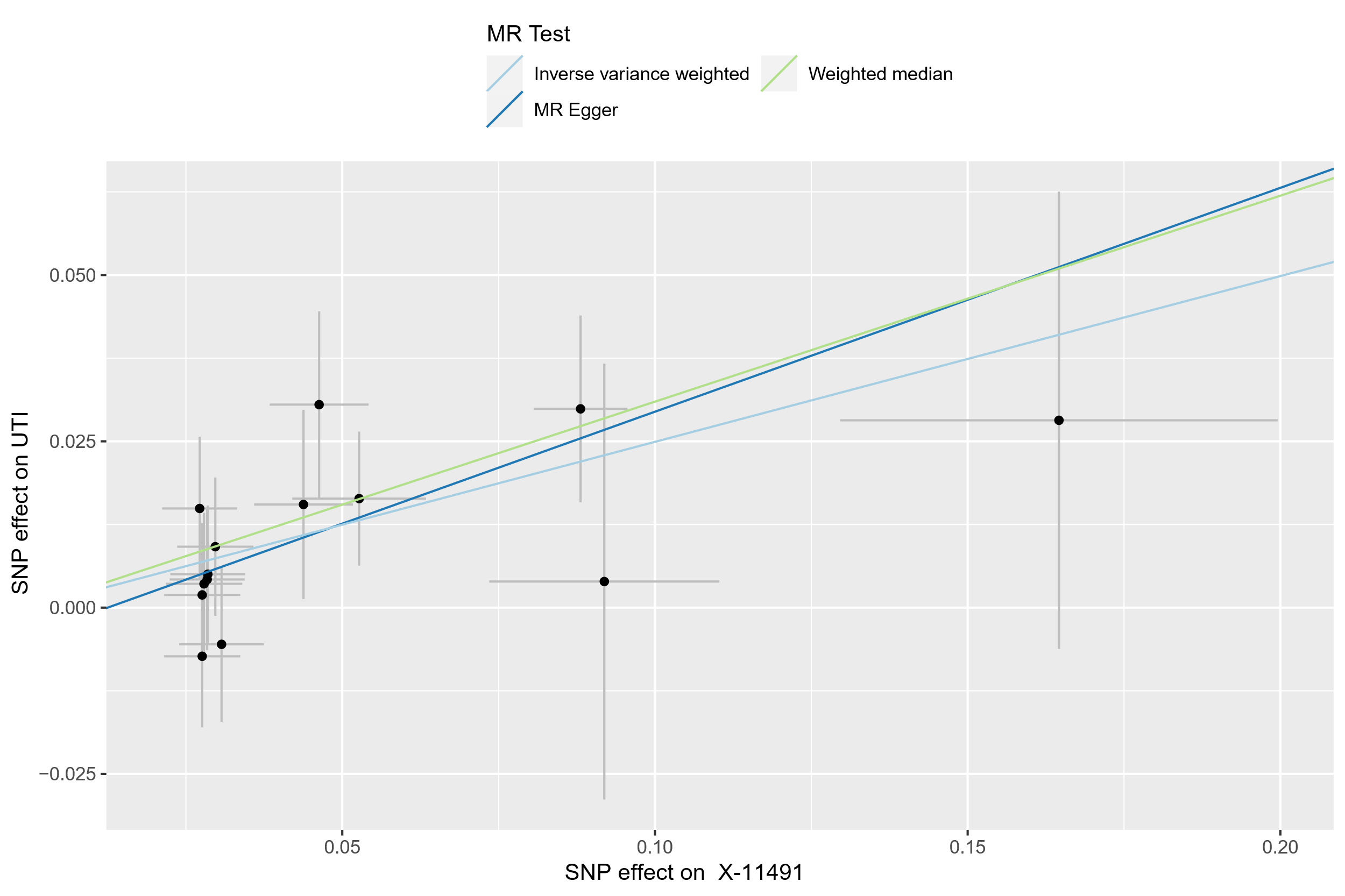

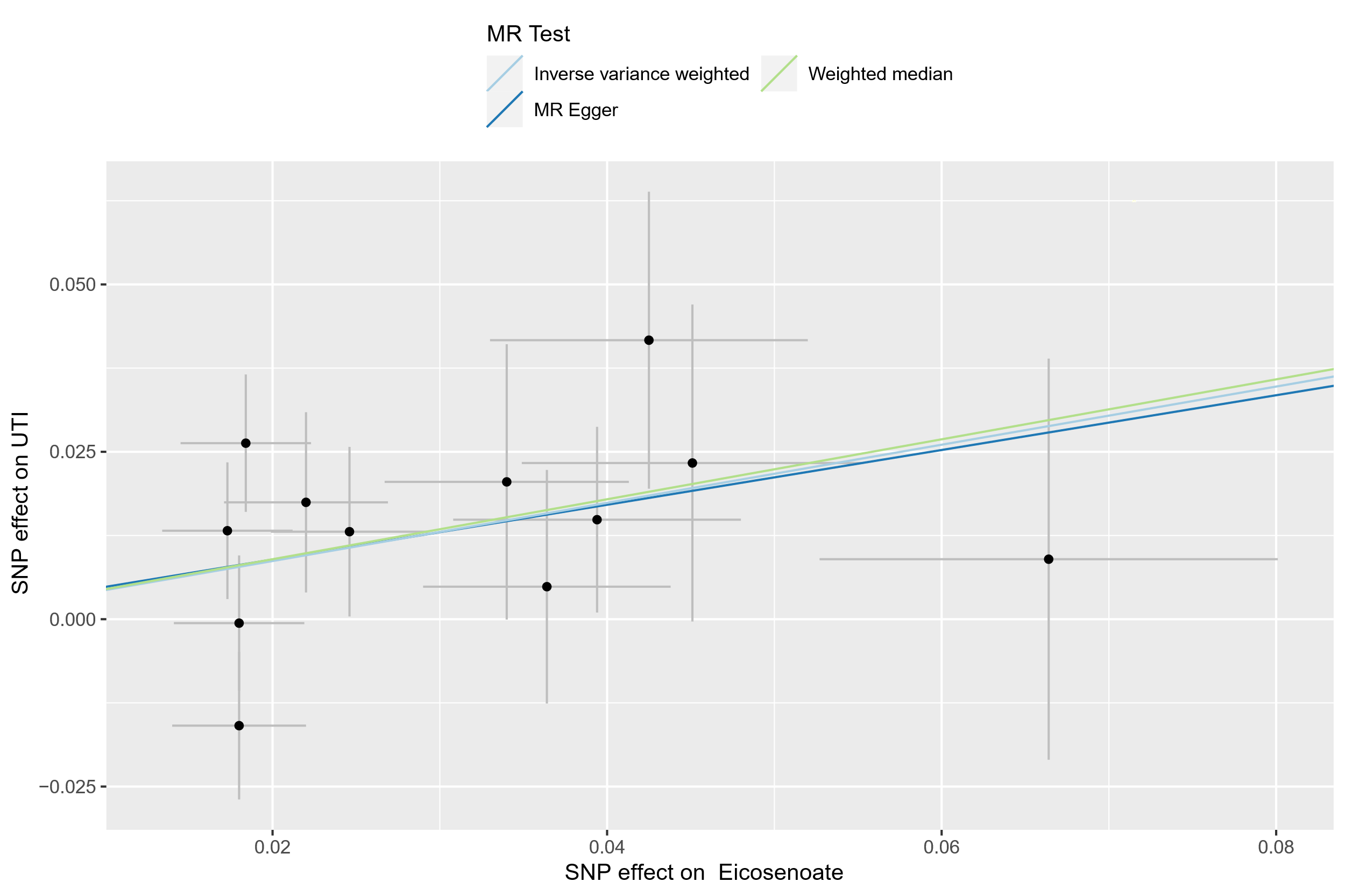


**Supplement Figure-1:** Scatterplot for the significant Mendelian randomization (MR) association (FDR < 0.05) between metabolites and 4 types of infection phenotypes (sepsis, pneumonia, URTI, and UTI). SNP, single nucleotide polymorphism; URTI, upper respiratory tract infection; UTI, urinary tract infection.


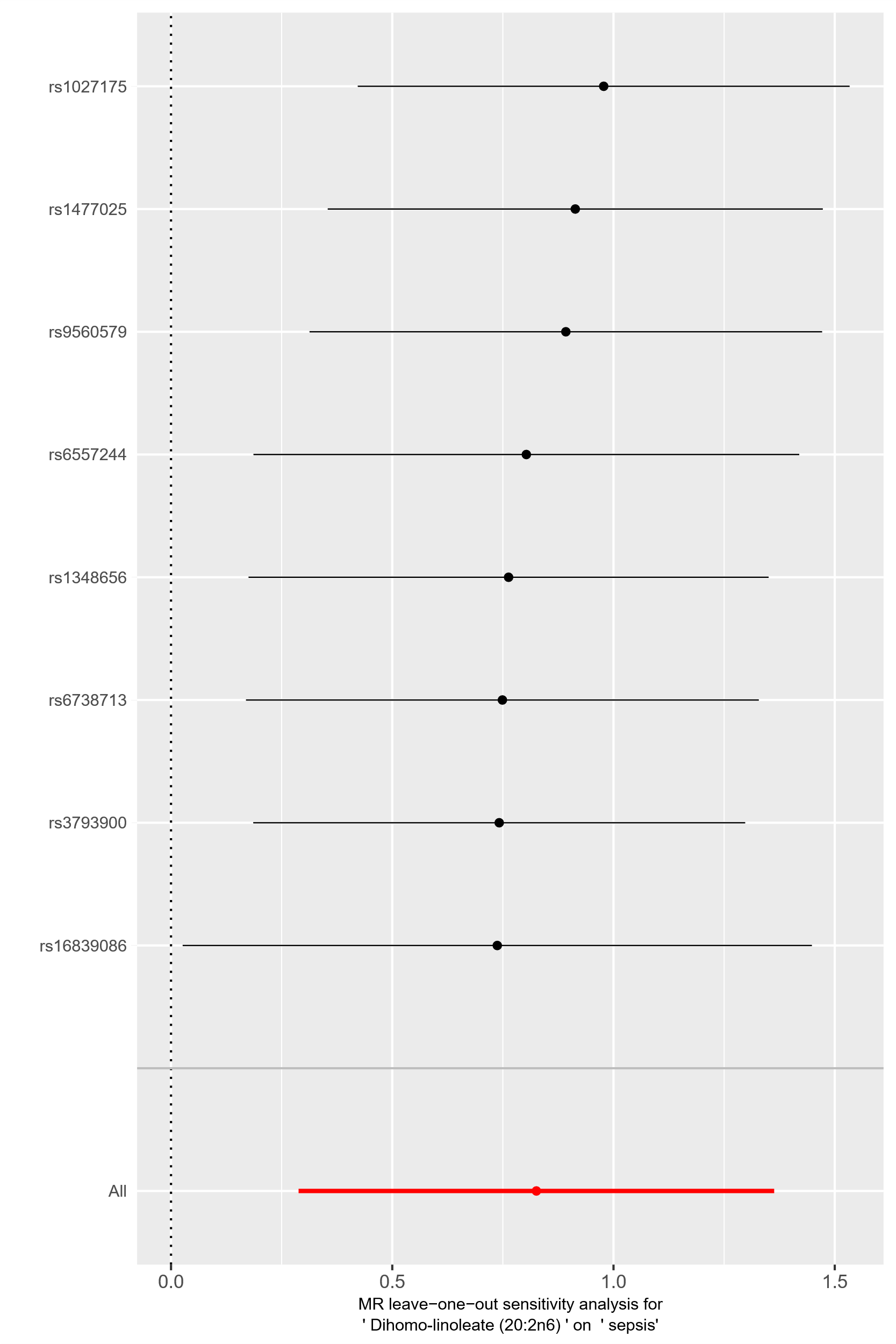

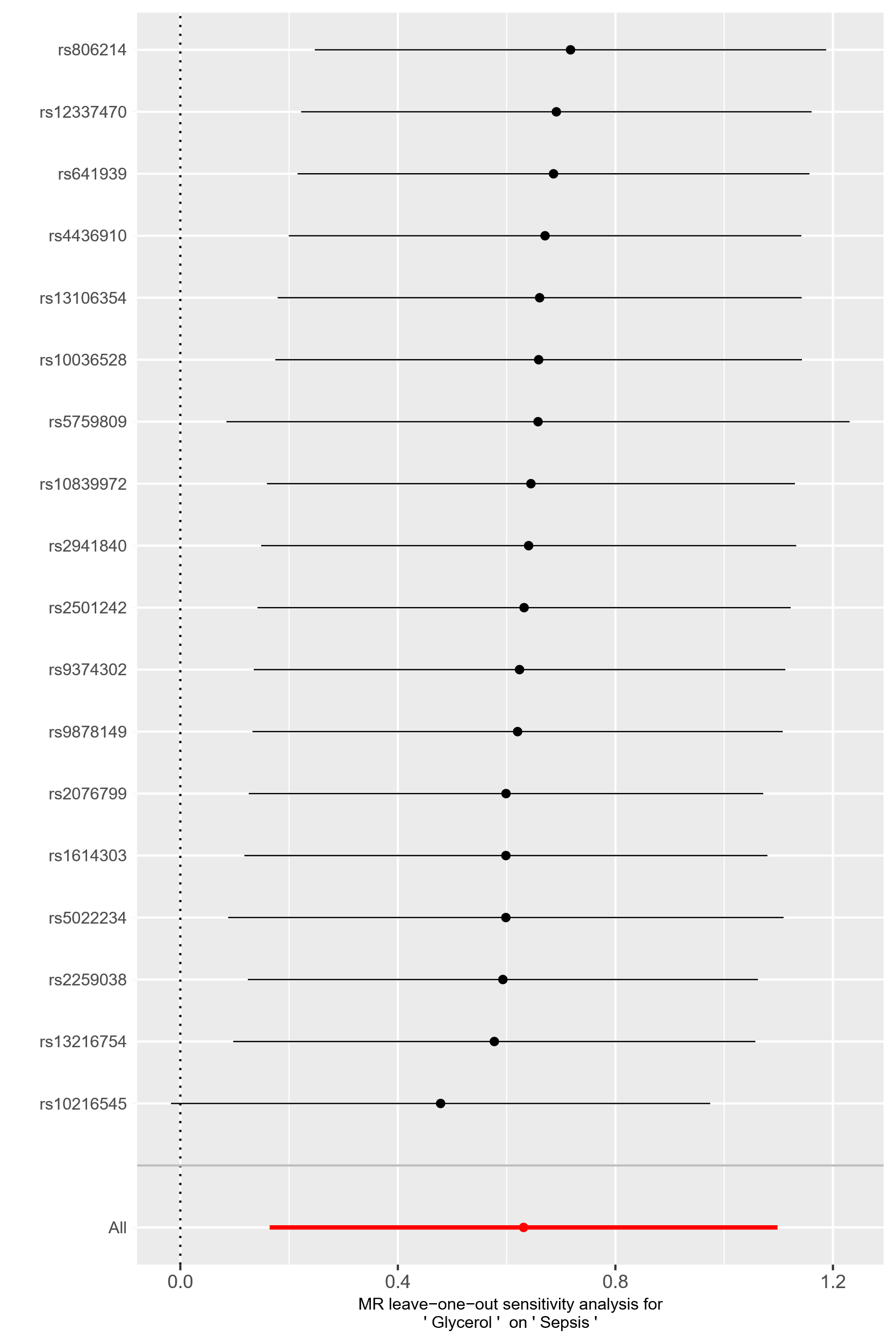

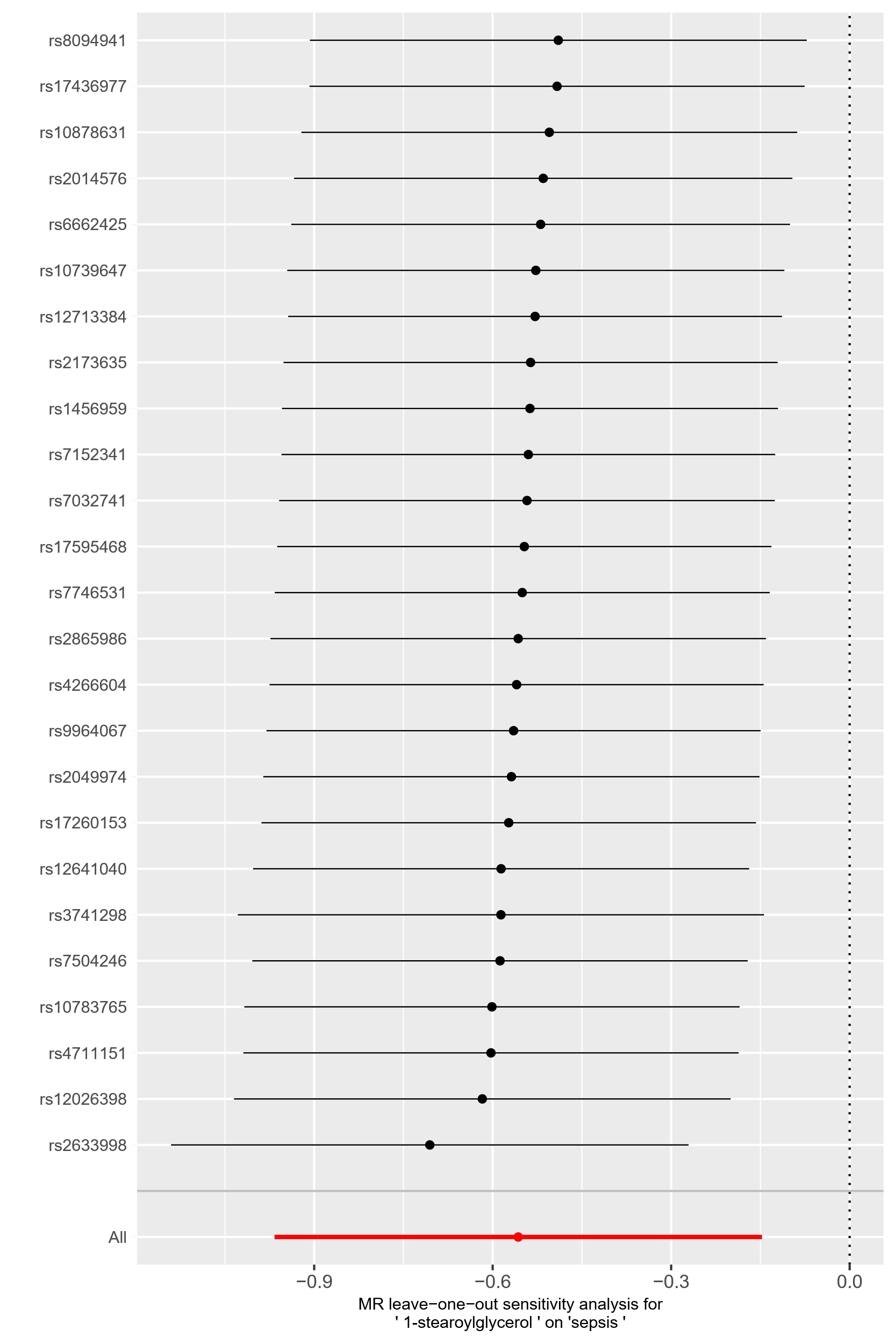

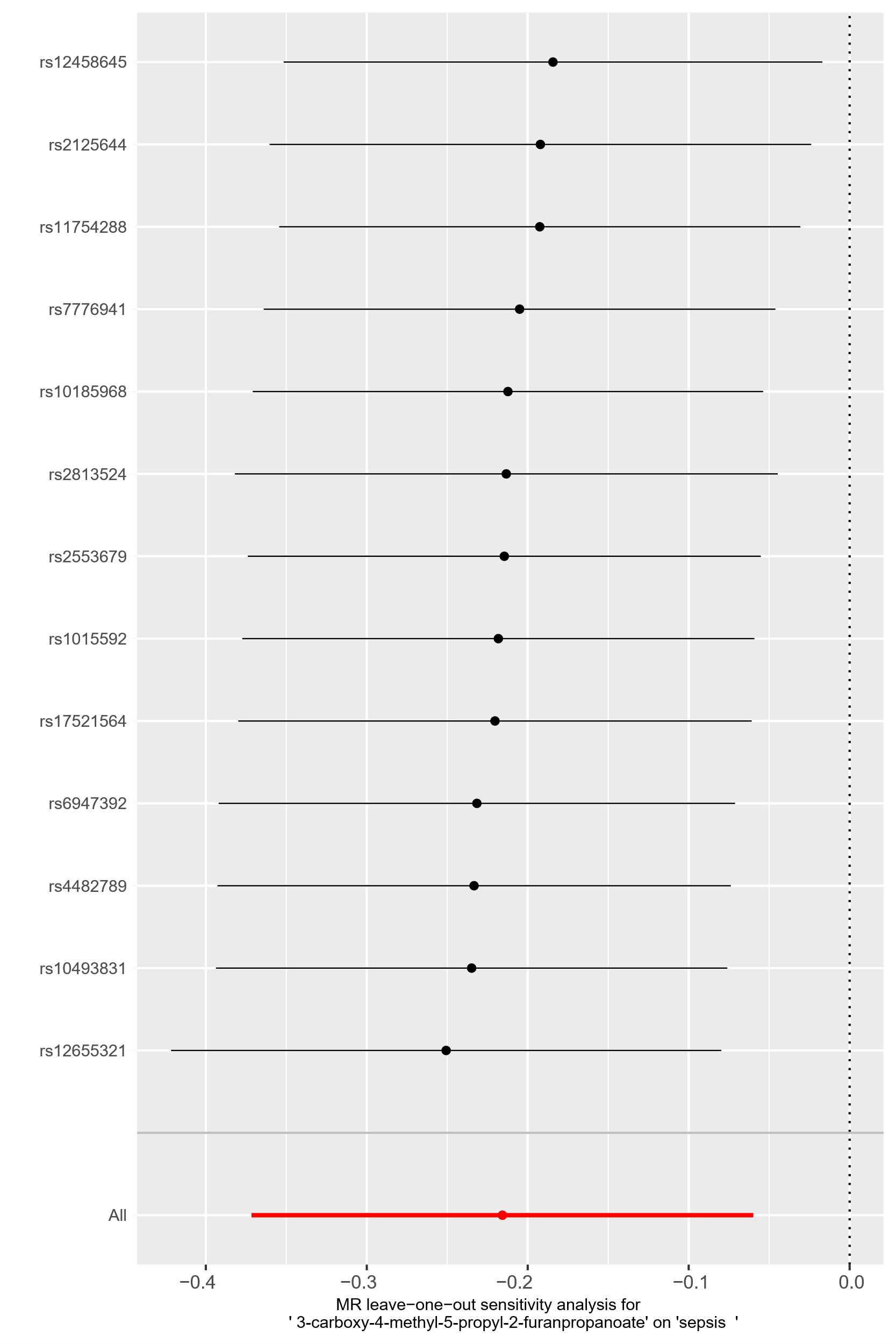

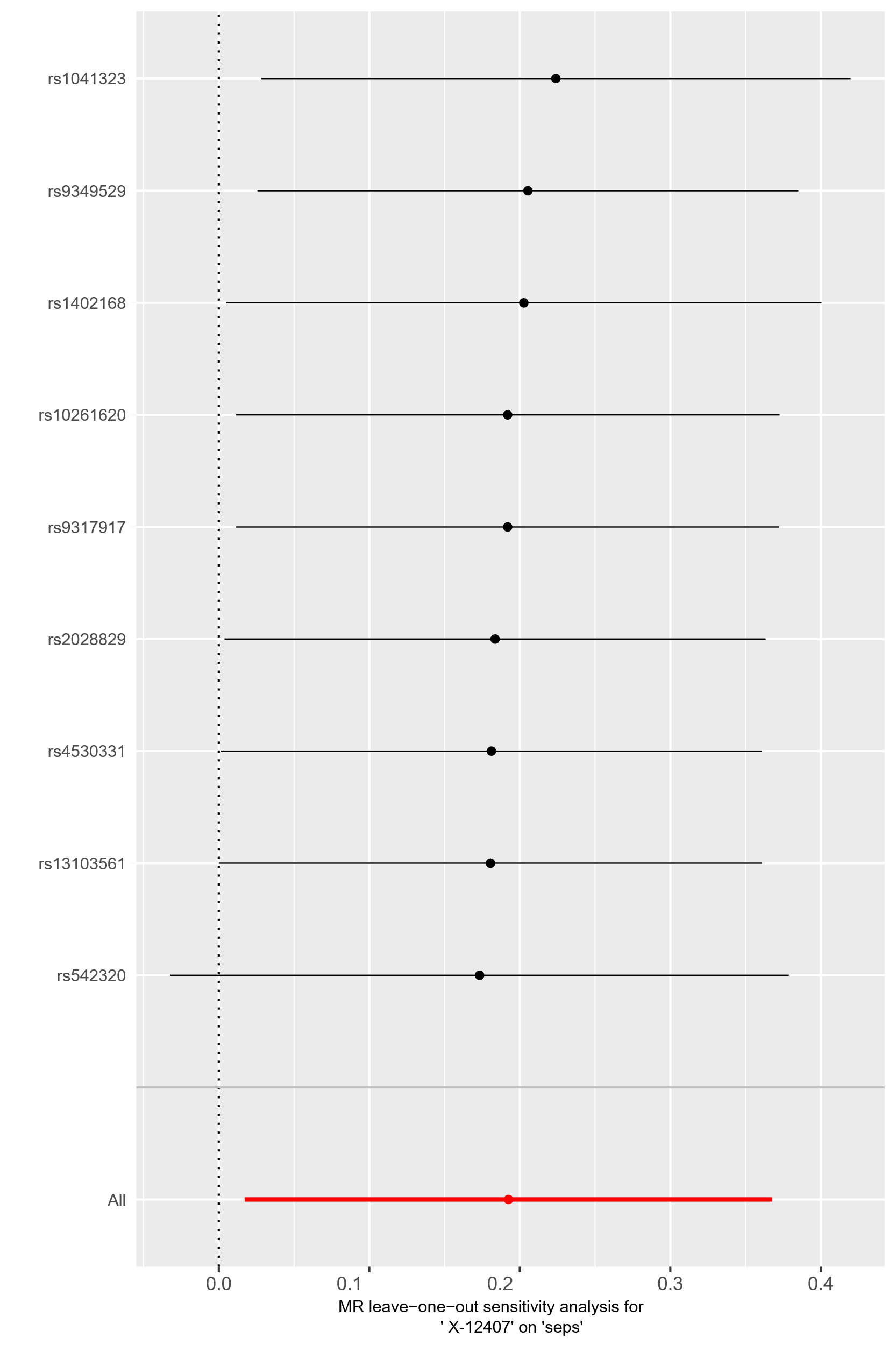

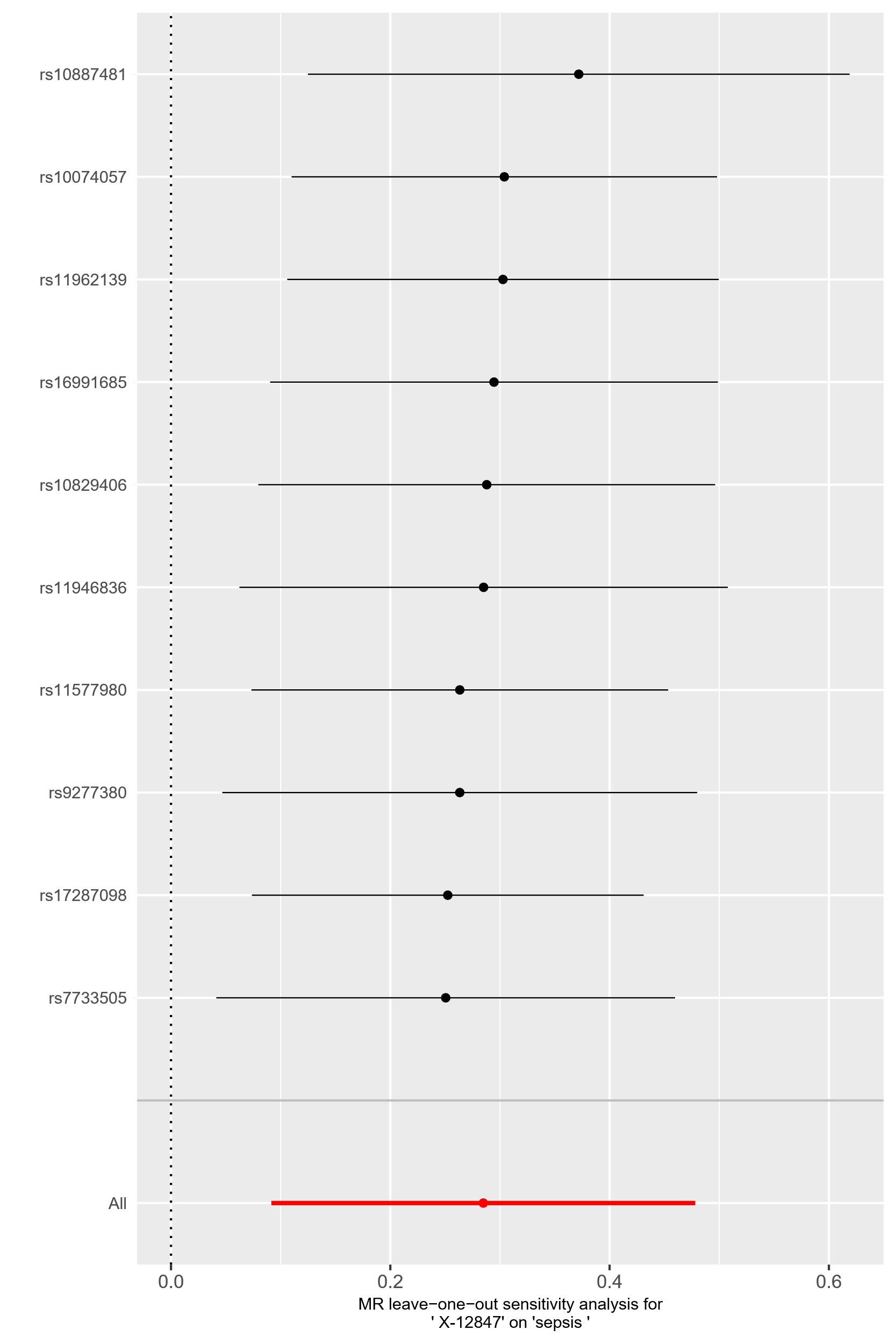

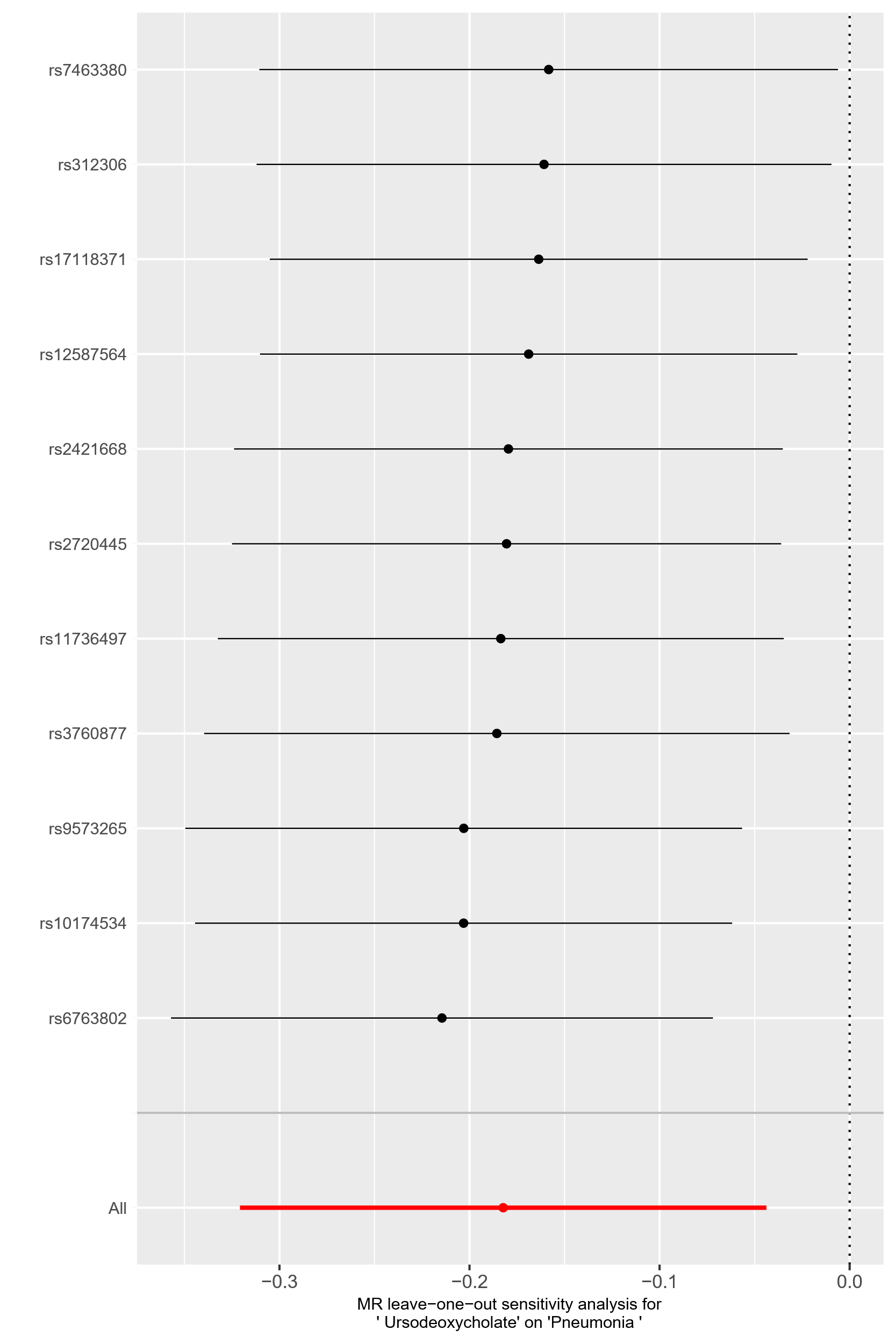

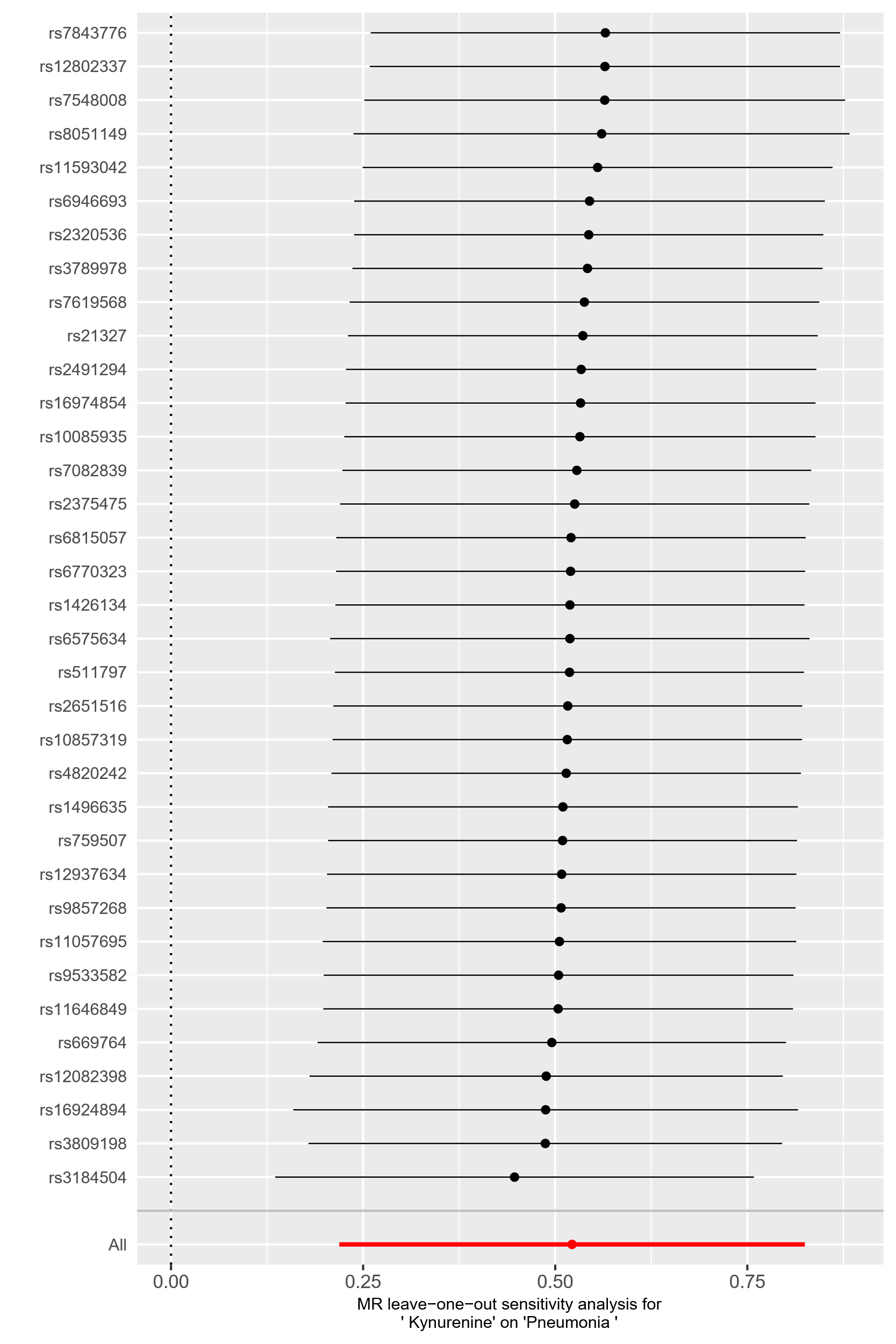

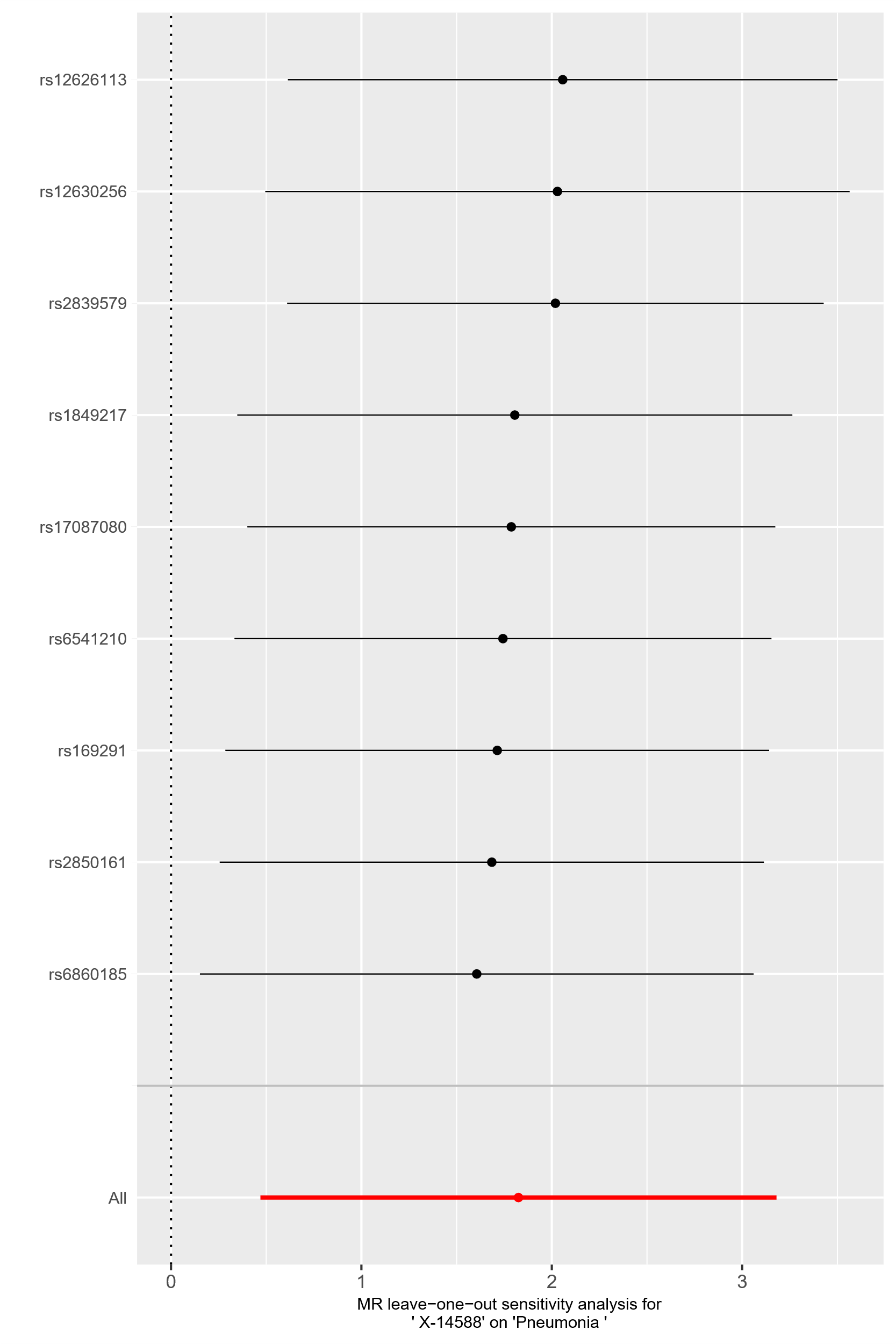

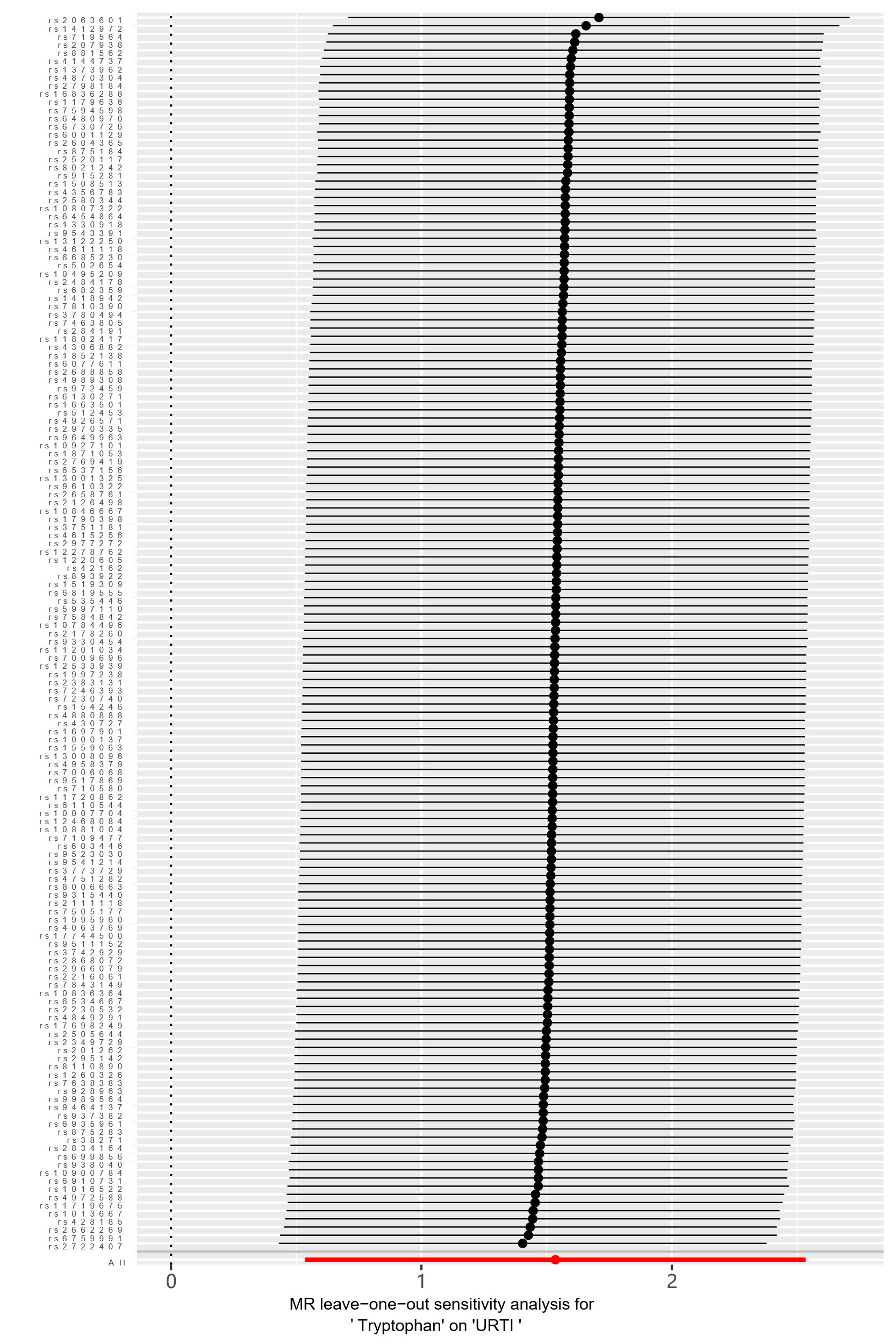

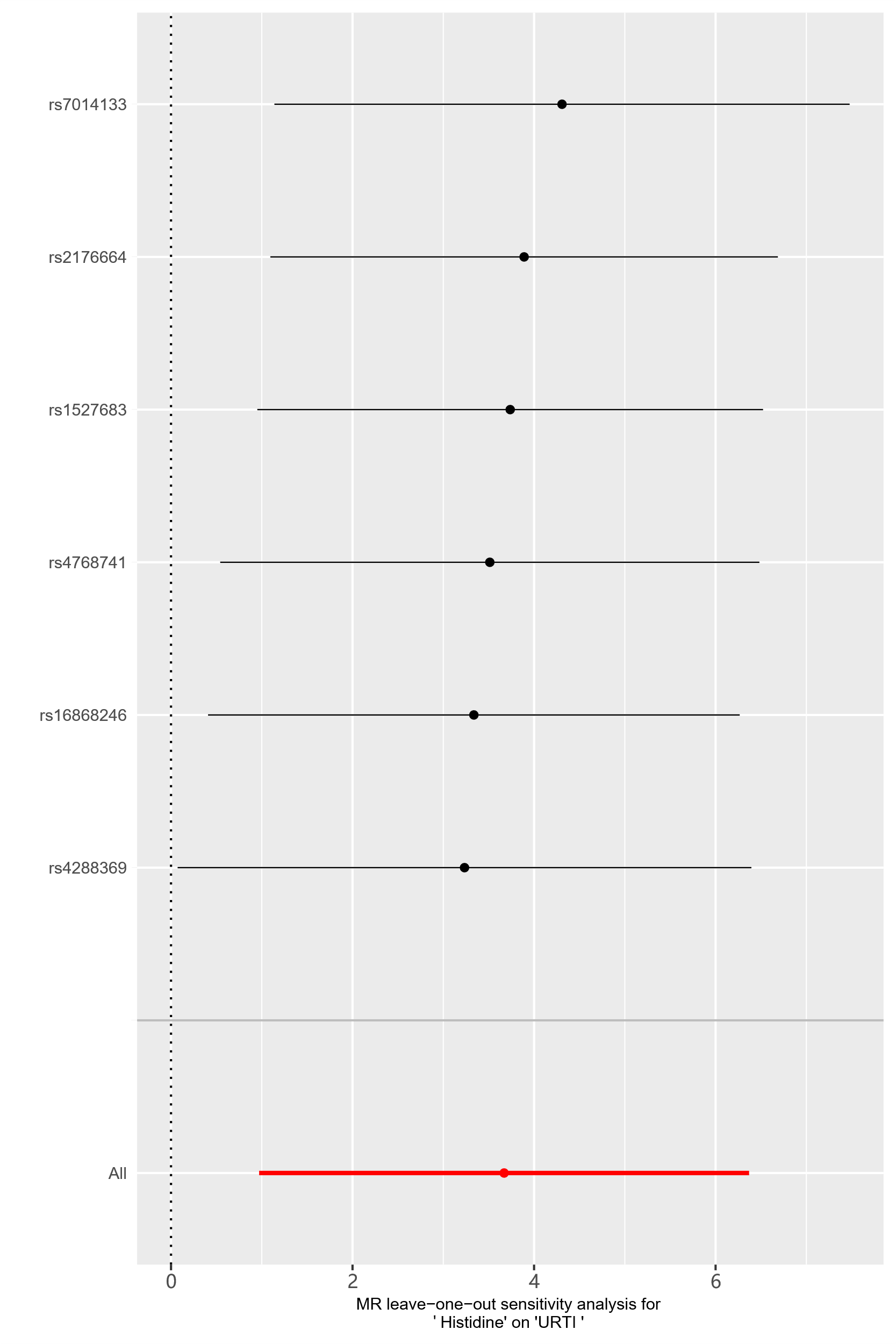

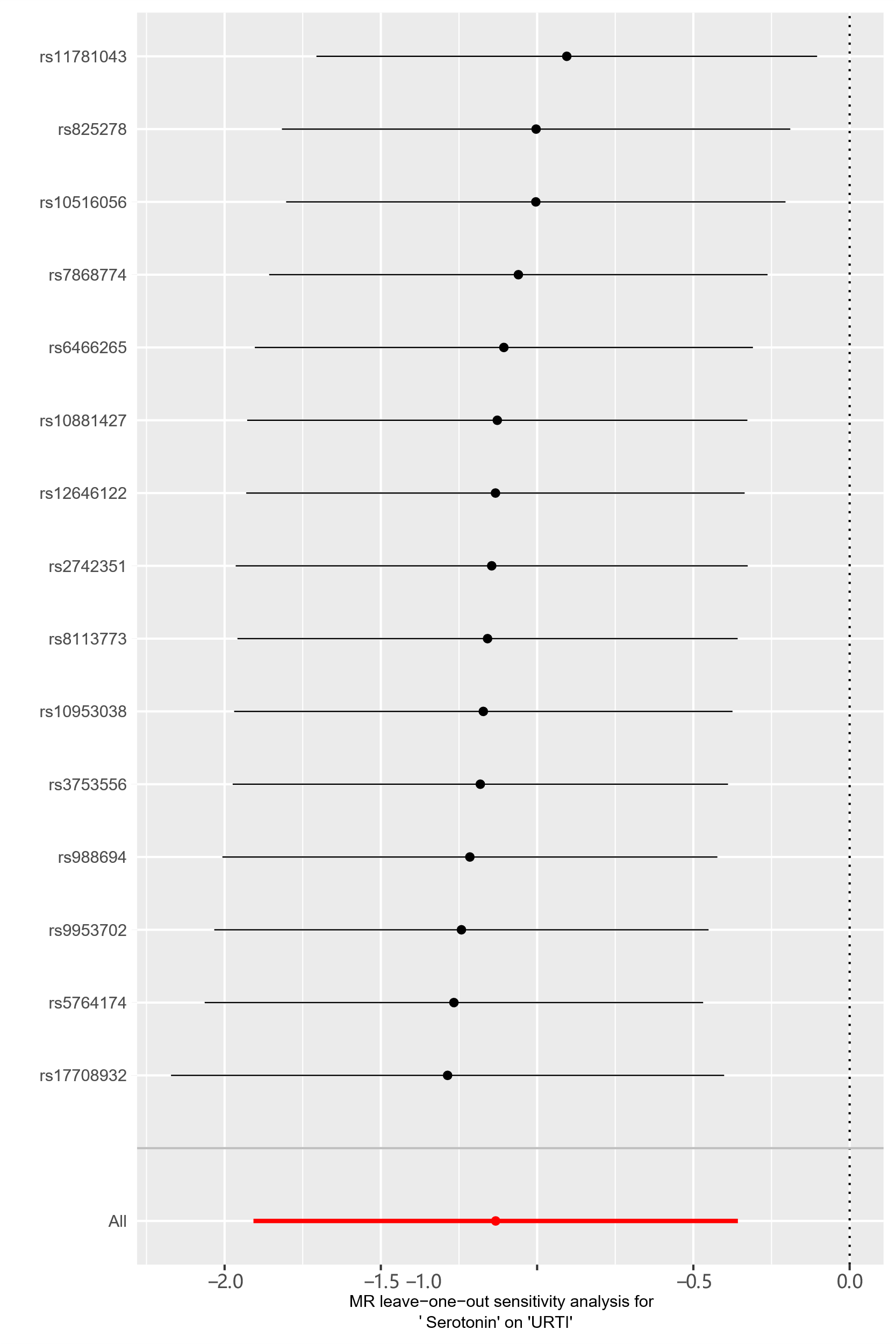

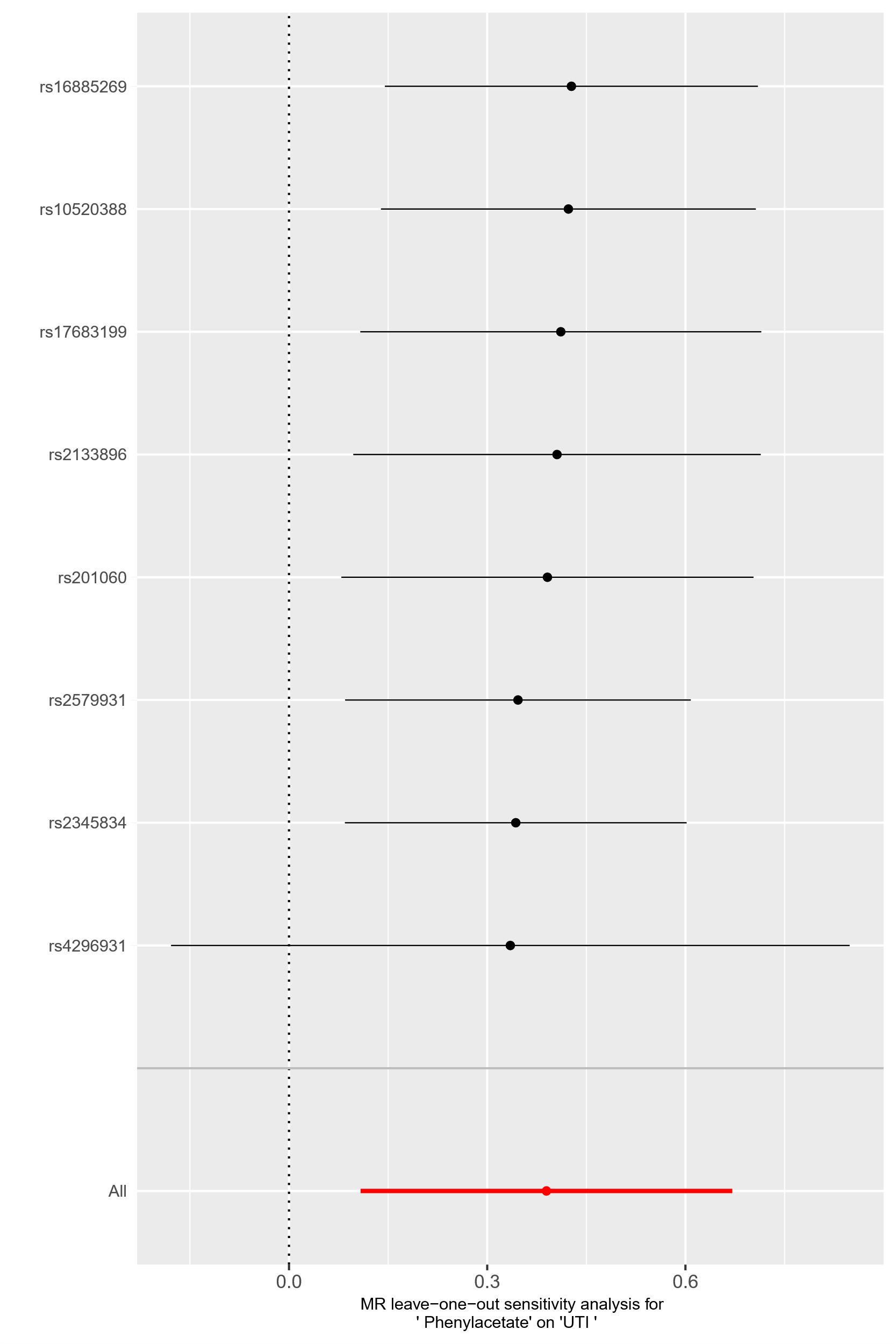

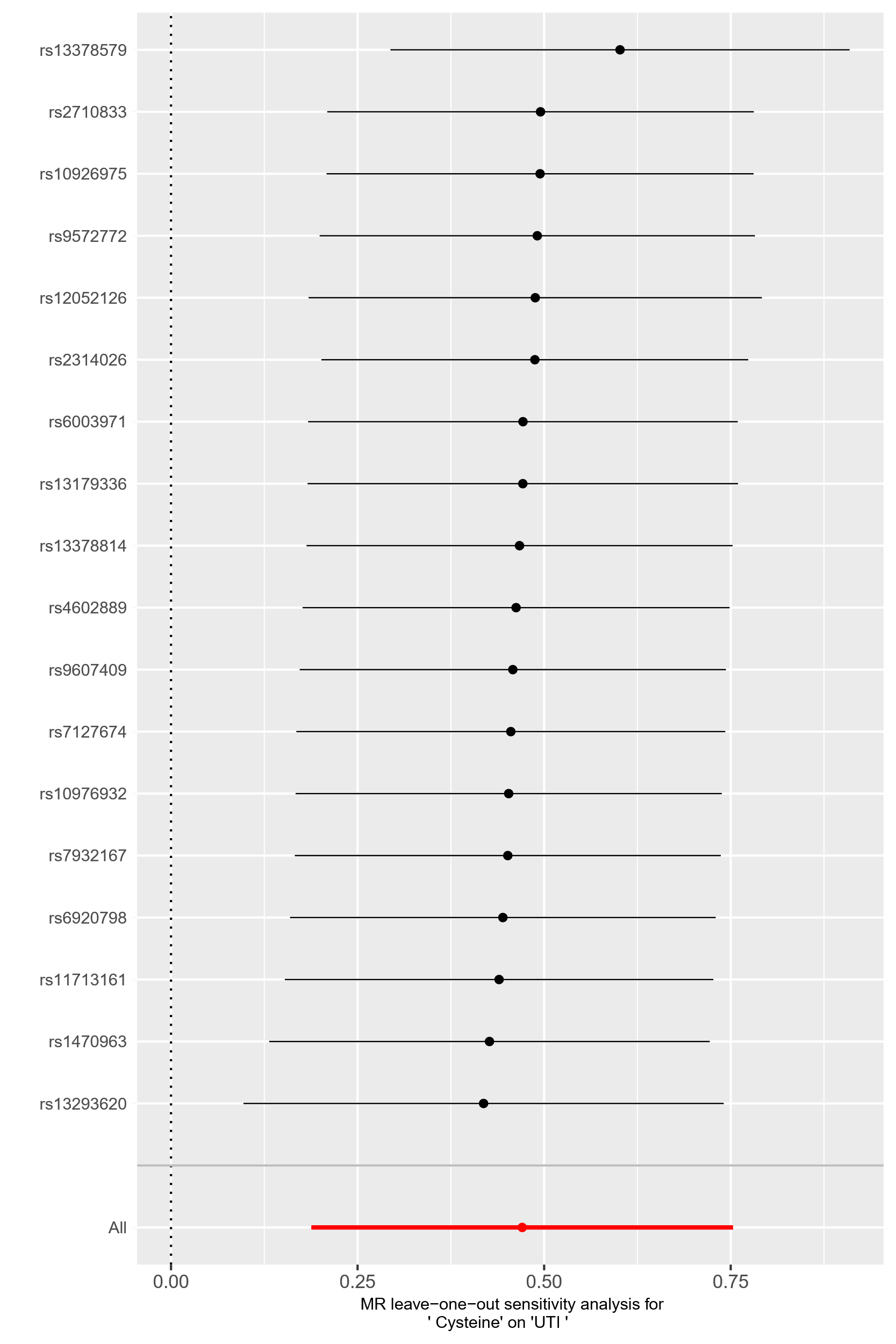

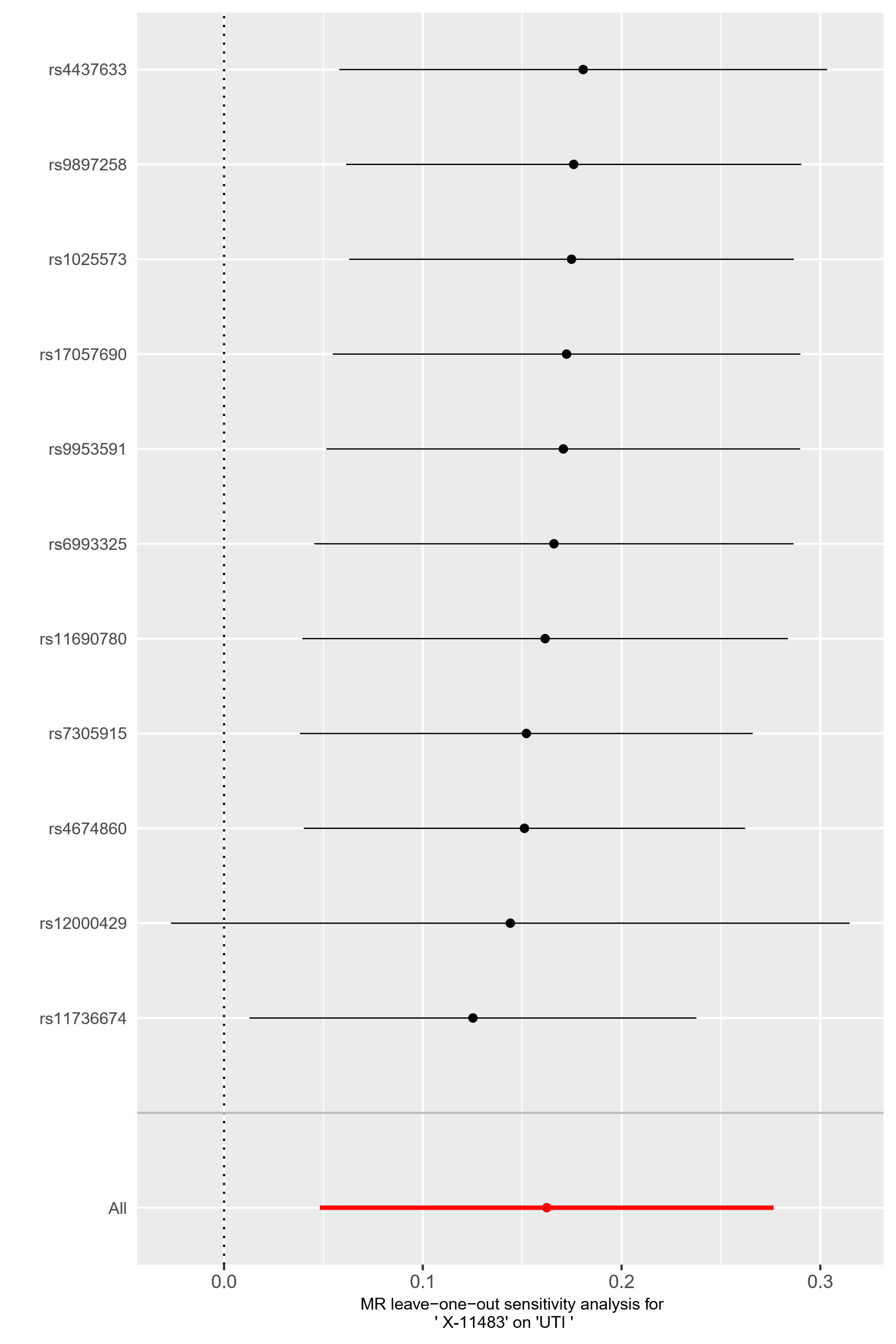

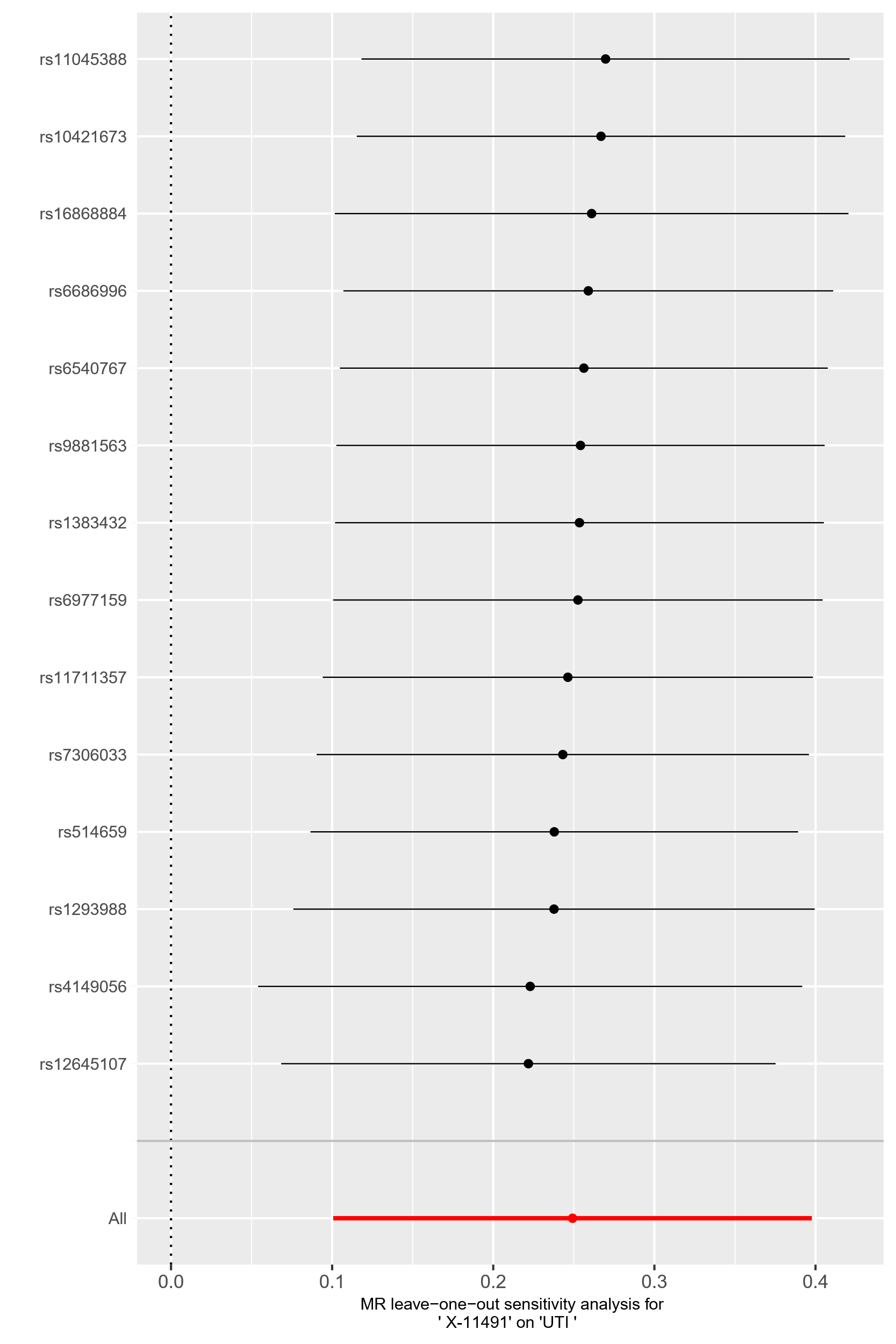

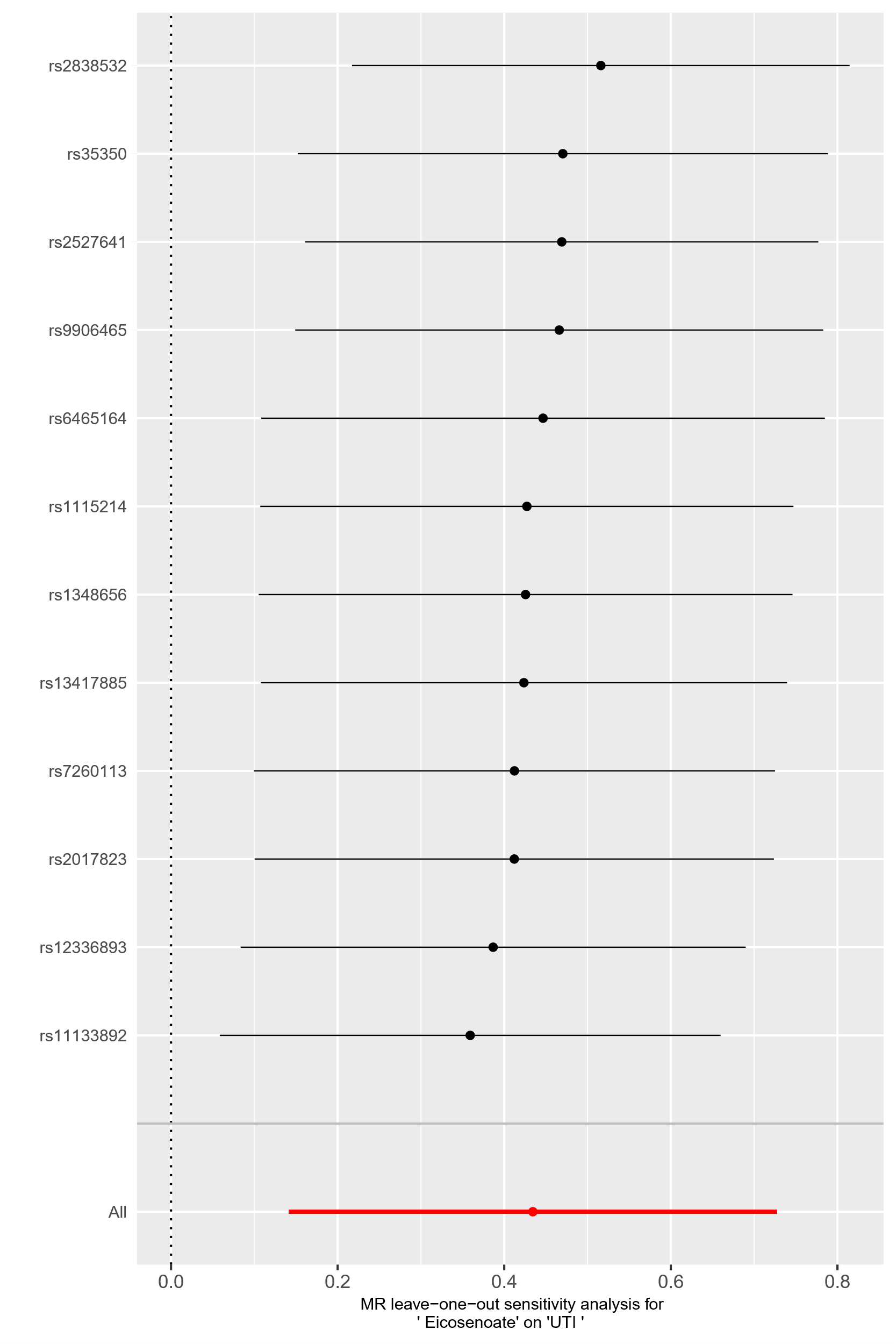


**Supplement Figure-2：** Forest plots for the Mendelian randomization (MR) leave-one-out analysis of the significant inverse variance weighted (IVW) estimates. URTI, upper respiratory tract infection; UTI, urinary tract infection.


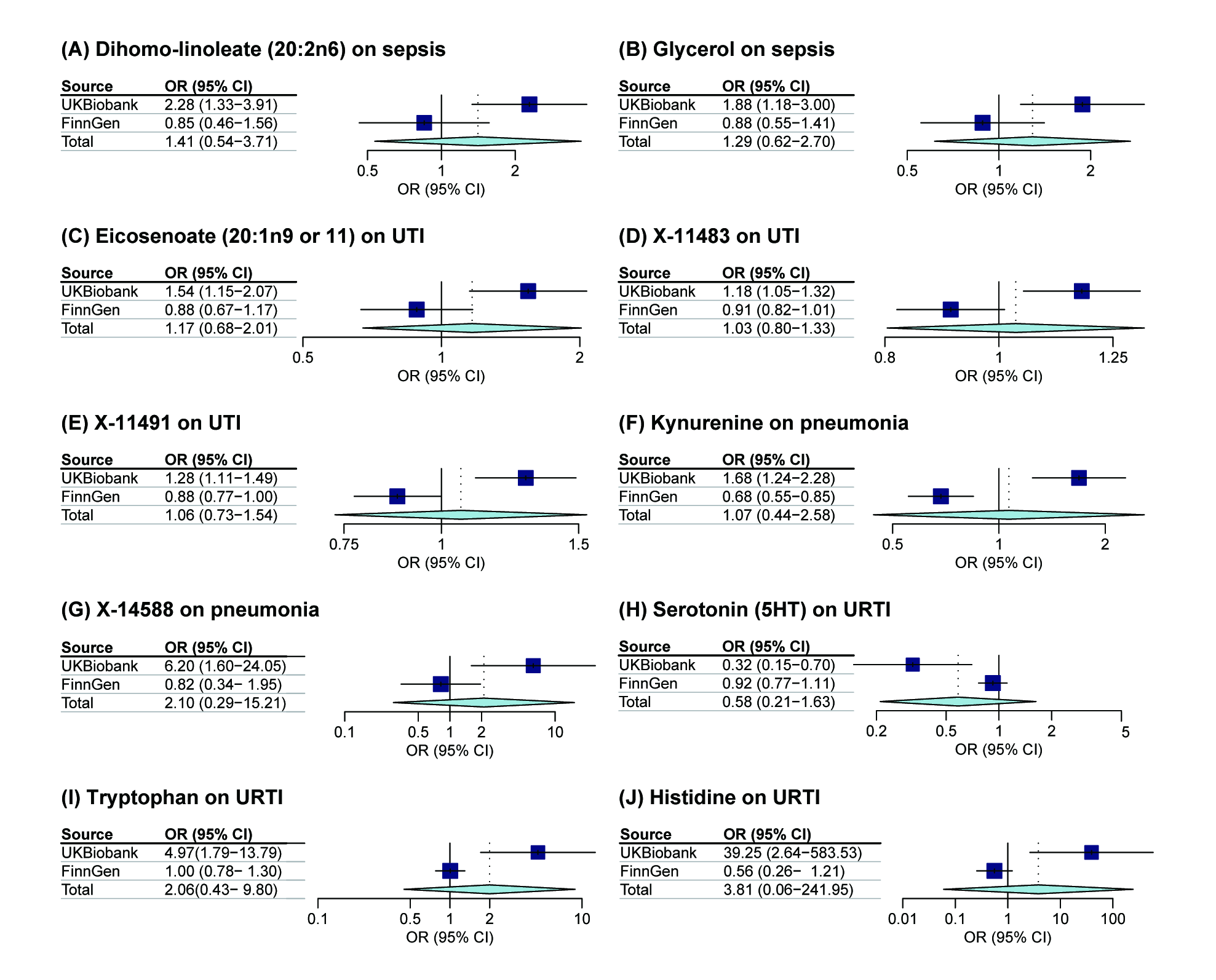


**Supplement Figure-3** Meta‑analysis of the causal associations between metabolites and 4 types of infection phenotypes (sepsis, pneumonia, URTI, and UTI). OR, odds ratio; CI, confidence interval; URTI, upper respiratory tract infection; UTI, urinary tract infection.
